# NXP800 Activates the Unfolded Protein Response, Altering AR and E2F Function to Impact Castration-Resistant Prostate Cancer Growth

**DOI:** 10.1158/1078-0432.CCR-24-2386

**Published:** 2025-01-09

**Authors:** Jonathan Welti, Denisa Bogdan, Ines Figueiredo, Ilsa Coleman, Juan Jiménez Vacas, Kate Liodaki, Franziska Weigl, Lorenzo Buroni, Wanting Zeng, Ilona Bernett, Claudia Bertan, Theodoros I. Roumeliotis, Amandeep Bhamra, Jan Rekowski, Bora Gurel, Antje J. Neeb, Jian Ning, Dapei Li, Veronica S. Gil, Ruth Riisnaes, Susana Miranda, Mateus Crespo, Ana Ferreira, Nina Tunariu, Elisa Pasqua, Nicola Chessum, Matthew Cheeseman, Robert te Poele, Marissa Powers, Suzanne Carreira, Jyoti Choudhary, Paul Clarke, Udai Banerji, Amanda Swain, Keith Jones, Wei Yuan, Paul Workman, Peter S. Nelson, Johann S. de Bono, Adam Sharp

**Affiliations:** 1 The Institute of Cancer Research, London, United Kingdom.; 2 Fred Hutchinson Cancer Center, Seattle, Washington.; 3 The Royal Marsden NHS Foundation Trust, London, United Kingdom.; 4 Centre for Cancer Drug Discovery at The Institute of Cancer Research, London, United Kingdom.

## Abstract

**Purpose:**

Advanced prostate cancer is invariably fatal, with the androgen receptor (AR) being a major therapeutic target. AR signaling inhibitors have improved overall survival for men with advanced prostate cancer, but treatment resistance is inevitable and includes reactivation of AR signaling. Novel therapeutic approaches targeting these mechanisms to block tumor growth is an urgent unmet clinical need. One attractive strategy is to target heat shock proteins (HSP) critical to AR functional activity.

**Experimental Design:**

We first did transcriptome analysis on multiple castration-resistant prostate cancer (CRPC) cohorts to correlate the association between the Gene Ontology cellular response to heat gene expression signature and overall survival. Next, we analyzed the impact of targeting the heat shock factor 1 (HSF1) pathway, with an inhibitor in clinical development, namely, NXP800 (formerly CCT361814), in models of treatment-resistant prostate cancer. Finally, we confirmed our mechanistic and phenotypic findings using an NXP800-resistant model and an *in vivo* model of CRPC.

**Results:**

We report that in multiple CRPC transcriptome cohorts, the Gene Ontology cellular response to heat gene expression signature associates with AR signaling and worse clinical outcome. We demonstrate the effects of targeting the HSF1 pathway, central to cellular stress, with an inhibitor in clinical development, namely, NXP800, in prostate cancer. Targeting the HSF1 pathway with the inhibitor NXP800 decreases HSP72 expression, activates the unfolded protein response, and inhibits AR- and E2F-mediated activity, inhibiting the growth of treatment-resistant prostate cancer models.

**Conclusions:**

Overall, NXP800 has antitumor activity against treatment-resistant prostate cancer models, including molecular subtypes with limited treatment options, supporting its consideration for prostate cancer–specific clinical development.

**Translational Relevance**

Advanced prostate cancer is lethal, and the androgen receptor (AR) remains a major therapeutic target. AR signaling inhibitors have improved overall survival for men with advanced prostate cancer, but treatment resistance is inevitable. The development of new treatments that abrogate key signaling pathways required for the development and progression of castration-resistant prostate cancer, through novel mechanisms of action, remains an urgent unmet clinical need. Herein, we demonstrate that NXP800, a heat shock factor 1 pathway inhibitor in clinical development, activates the unfolded protein response and inhibits AR- and E2F-mediated transcription as well as the growth of treatment-resistant prostate cancer models. Taken together, this therapeutic strategy merits clinical evaluation in men suffering from metastatic castration-resistant prostate cancer.

## Introduction

Prostate cancer is the most common cancer and the second highest cause of cancer-related death in men in the Western world and is increasing in incidence (1). The androgen receptor (AR) remains the main oncogenic driver and a major therapeutic target for advanced prostate cancer (2–5). Despite the development of multiple AR signaling inhibitors (ARSI, including abiraterone, enzalutamide, darolutamide, and apalutamide) that have improved the clinical outcome for patients with advanced disease, treatment resistance is inevitable and advanced prostate cancer remains fatal (5). The development of treatment resistance is commonly associated with reactivation of AR signaling which, in part, is driven by AR amplifications, AR-activating point mutations, and constitutively active AR splice variants, of which AR splice variant-7 (AR-V7) is the most clinically relevant (6–14). Currently approved therapies, and many of those in clinical development, elicit their mechanism of action through the AR ligand–binding domain and therefore will have limited activity against these mechanisms of resistance (5–16). Therefore, the development of innovative therapeutic strategies that abrogate persistent AR signaling through novel mechanisms of action is an urgent unmet clinical need in prostate cancer medicine.

One attractive strategy is to target co-regulators, such as the heat shock proteins (HSP), critical to AR activity (17–28). HSPs are a family of molecular chaperones that regulate protein homeostasis in response to cellular stress (29). Upon exposure to cellular stress, the transcription factor heat shock factor 1 (HSF1) upregulates the expression of multiple HSPs (including HSP90, HSP72, and HSP27) to protect the cell from proteotoxic stress (29–33). Importantly, HSP90, HSP72, and HSP27 have all been implicated in fueling AR signaling in prostate cancer (17–27). First, blocking HSP90 function in prostate cancer decreases AR nuclear translocation and enhances its degradation, leading to decreased transcriptional activity and proliferation (22–26). In addition, HSP90 inhibition decreases AR-V7 expression by regulating RNA processing, inhibiting the growth of treatment-resistant prostate cancer models (18). Second, HSP72 is critical for AR and AR-V7 stability, with HSP72 inhibition decreasing the growth of enzalutamide-resistant prostate cancer models (17, 19, 27, 28). Third, inhibition of HSP27 leads to AR and AR-V7 degradation with associated growth inhibition of treatment-resistant prostate cancer models (20, 21). Taken together, targeting the HSP family of molecular chaperones is a valid therapeutic approach to overcome persistent AR signaling in advanced prostate cancer, although concerns remain about the therapeutic window and tolerability of such HSP-targeting strategies (30, 34). Nevertheless, the oral HSP90 inhibitor pimitespib has been approved in Japan for gastrointestinal stromal tumor (35).

The pursuit of developing novel approaches targeting HSPs has resulted in the identification of inhibitors of the HSF1 pathway as an alternative therapeutic strategy (30, 36, 37). However, the majority of these inhibitors have not been optimized for selectivity or drug-like pharmaceutical properties, have limited information on potential mechanism of action, and are not at the clinical stage of development (36, 37). Therefore, although inhibition of the HSF1 pathway has been investigated in advanced prostate cancer models with promising antitumor activity, clinical translation remains challenging (38, 39). By contrast, CCT251236, which we partially optimized from a compound that we identified in a cell-based assay for inhibition of HSF1-mediated HSP72 induction following pharmacologic inhibition of HSP90, suppresses HSP72 and HSP27 transcription and shows promising activity in models of human multiple myeloma and ovarian adenocarcinoma cell line–derived xenografts (40, 41). Further multiparameter pharmaceutical optimization has led to our design of NXP800 (formerly CCT361814), which is currently undergoing clinical evaluation (NCT05226507), providing an opportunity to clinically translate this therapeutic strategy (42, 43).

Herein we present the first evaluation of NXP800 in models of advanced prostate cancer. We report that a higher Gene Ontology (GO) cellular response to heat gene expression signature in advanced prostate cancer tissue biopsies associates with increased AR signaling and worse overall survival (OS). In addition, we demonstrate that NXP800 reduces HSP72 expression and decreases AR signaling, reduces AR and AR-V7 protein expression as well as their DNA binding and transactivation, and also inhibits the growth of treatment-resistant prostate cancer models. Moreover, we report that NXP800 inhibits the growth of AR-independent prostate cancer models, indicating that its antitumor activity is not only mediated through abrogation of AR signaling. Consistent with this, we demonstrate that NXP800 activates the unfolded protein response (UPR) and inhibits key signaling pathways, including AR- and E2F-mediated transcription, which are implicated in the development and progression of castration-resistant prostate cancer (CRPC), thus providing further insights into the mechanism of NXP800-mediated antitumor activity. Finally, these mechanistic and phenotypic findings were confirmed using an NXP800-resistant model and an *in vivo* model of CRPC.

Taken together, our findings show that NXP800 demonstrates antitumor activity in treatment-resistant prostate cancer models, acting through a novel mechanism of action that has the potential to provide a new therapeutic strategy for hard-to-treat prostate cancer tumors with persistent AR signaling and high E2F activity, supporting its consideration for prostate cancer–specific clinical development.

## Materials and Methods

### Cell lines and compounds

All cell lines used in this study were grown in the suppliers’ recommended media at 37°C in 5% CO_2_ and are detailed in Supplementary Table S1. Parental cell lines were tested for *Mycoplasma* using the VenorGeM OneStep PCR Kit (Cambio) and short tandem repeat profiled using the cell authentication service by Eurofins Medigenomix. All compounds used are detailed in Supplementary Table S2. These included the HSF1 pathway inhibitor clinical candidate NXP800 (Supplementary Fig. S1A) and the closely matched regioisomer CCT365248 used as an inactive control compound (Supplementary Fig. S1B), which were both synthesized in the Centre for Cancer Drug Discovery, Division of Cancer Therapeutics, The Institute of Cancer Research (43).

### Plasmids

The plasmids used in this study were the pReceiver-M13 vector carrying a C-terminal fusion FLAG-tag (GeneCopoeia) containing either an empty cassette as control (CONTROL-FLAG) or full-length AR (AR-FL, AR-FL-FLAG). An AR-V7 C-terminal fusion FLAG-tag (AR-V7-FLAG) was generated from the corresponding AR-FL-FLAG plasmid through restriction enzyme digest (XhoI, BSTE1I; New England Biolabs) and ligation techniques as previously described (44). The resulting plasmids were verified by sequencing (Beckman Coulter Genomics). The PSA-ARE3-luciferase reporter plasmid has been previously described (45).

### Immunoprecipitation–mass spectrometry

PC3 cells were transfected with 0.5 μg/mL CONTROL-FLAG, AR-FL-FLAG, or AR-V7-FLAG using X-tremeGENE (Roche) as per the manufacturer’s recommendations. After 48 hours, whole-cell lysates were obtained using Pierce Immunoprecipitation (IP) Lysis Buffer (Thermo Fisher Scientific). Protein extracts (about 1.5 mg of total protein) were precleared by protein A/G Plus-Agarose (Thermo Fisher Scientific), and immune complexes were collected by incubation with 1 μg FLAG antibody (Thermo Fisher Scientific, Cat. #MA1-91878, RRID: AB_1957945) and protein A/G Plus-Agarose beads overnight at 4°C. Bead/sample complexes were collected by centrifugation and washed first in standard lysis buffer and then in 50 mmol/L triethylammonium bicarbonate solution (Sigma-Aldrich). Bound proteins were then eluted using 5% formic acid and then dried *in vacuo* without heating. Proteins were thoroughly redissolved in 5% acetonitrile/50 mmol/L triethylammonium bicarbonate solution before being reduced with 5 mmol/L tris(2-carboxyethyl)phosphine. Free cysteines were alkylated with 10 mmol/L 2-choloroacetamide and digested with trypsin (Promega). The digestion was quenched with neat formic acid after 4 hours. LC/MS analysis was performed on an LTQ Orbitrap Velos (Thermo Fisher Scientific) with collision-induced dissociation (CID) fragmentation and ion trap detection. Tandem mass spectra were analyzed with Mascot (version 2.3.02; RRID: SCR_014322) for peptide and protein identification using a Swiss-Prot FASTA file containing 20,305 *Homo sapiens* entries. Mascot was searched with a fragment ion mass tolerance of 0.6 Da and a parent ion tolerance of 5 ppm. Gln->pyro-Glu of the n-terminus, oxidation of methionine, acetylation of the n-terminus, carbamidomethylation of cysteine, and phosphorylation of serine, threonine, and tyrosine were specified as variable modifications. Scaffold (Proteome Software Inc.) was used to validate MS/MS-based peptide and protein identifications. Peptide identifications were accepted if they could be established at >95.0% probability by the PeptideProphet algorithm (46). Protein identifications were accepted if they contained at least two identified peptides. Protein probabilities were assigned by the ProteinProphet algorithm (47).

### Transcriptome analyses of patients with CRPC

CRPC transcriptomes from the Prostate Cancer Foundation-Stand Up To Cancer (PCF-SU2C) cohort were downloaded and reanalyzed (2). CRPC transcriptomes from The Institute of Cancer Research-Royal Marsden Hospital (ICR-RMH) cohort were analyzed as previously described (48). Briefly, paired-end transcriptome sequencing reads for each of the PCF-SU2C (*n* = 159) and ICR-RMH (*n* = 95) cohorts were aligned to the human reference genome (GRCh37/hg19) using TopHat2 (v2.0.7; RRID: SCR_013035). Gene expression in fragments per kilobase of transcript per million mapped reads (FPKM) was calculated using Cufflinks (RRID: SCR_014597). CRPC transcriptomes from the University of Washington (UW) cohort were analyzed as previously described (3, 49). Briefly, sequencing reads were mapped to hg38 using STAR (v2.7.3a; RRID: SCR_004463). Gene-level abundance was quantitated using GenomicAlignments (RRID: SCR_024236) and transformed to log_2_ FPKM in edgeR (RRID: SCR_012802). Single-sample enrichment scores were calculated using gene set variation analysis with the *z*-score method and transcriptome-wide log_2_ FPKM values as the input (50). GO cellular response to heat gene expression signature was chosen for clinical analyses based on enrichment in VCaP prostate cancer cells following heat shock when screening for Molecular Signatures Database (MSigDB) pathways matching the search term “heat” and discounting the pathways that were negatively associated and those wherein none of the genes were differentially expressed after heat shock. The expression of each gene comprising the signature GO cellular response to heat (GO:0034605; Supplementary Table S3), Hallmark Androgen Response, Hallmark E2F targets, and previously described AR signatures (3, 14, 51, 52) was used to determine an aggregate expression *z*-score per patient, per signature. The Pearson correlation coefficient (*r*) and *P* value were calculated between the signature *z*-scores.

### Western blotting

Cell lines were lysed with RIPA buffer (Pierce) supplemented with protease inhibitor cocktail (Roche) and PhosSTOP phosphatase inhibitor mix (Roche). Experiments in which puromycin incorporation was used as a measure for protein translation had an additional step of 1 μmol/L puromycin treatment for 30 minutes prior to lysis. Castration-resistant VCaP prostate cancer cell line–derived mouse xenograft lysate was obtained by mechanical homogenization and reconstituted in RIPA buffer. Protein extracts (20 μg) were separated on 4% to 12% NuPAGE Bis-Tris gel (Invitrogen) by electrophoresis and subsequently transferred onto Immobilon-P polyvinylidene difluoride membranes of 0.45-μm pore size (Millipore). Details of primary antibodies used are provided in Supplementary Table S4. Chemiluminescence was detected on the ChemiDoc Touch Imaging System (Bio-Rad).

### *In vitro* cell line proliferation

*In vitro* proliferation of cell lines was measured using the CellTiter-Glo 2D Luminescent Cell Viability Assay (Promega) according to the manufacturer’s instructions, and luminescence was measured using Synergy HTX (BioTek).

### RNA extraction

Cell line RNA was extracted using the RNeasy Plus Mini Kit (QIAGEN) as per the manufacturer’s instructions. Castration-resistantVCaP prostate cancer cell line–derived mouse xenograft RNA was obtained by mechanical homogenization, reconstituted with RNeasy RLT buffer, passed through a QIAshredder tube (QIAGEN), and further processed with RNeasy Plus Mini Kit as per the manufacturer’s instructions.

### qRT-PCR

cDNA was synthesized using the RevertAid First Strand cDNA Synthesis Kit (Thermo Fisher Scientific). qPCR was carried out using a ViiA 7 Real-Time PCR System (Life Technologies) using the TaqMan Universal PCR Master Mix (Applied Biosystems). TaqMan probes (Thermo Fisher Scientific) used are listed in Supplementary Table S5. Fold change in RNA expression levels was calculated by the comparative CT method, using the formula 2^-^^ΔΔCt^. Results from all studies were normalized against the average of four housekeeping genes *RPLP0*, *GAPDH*, *B2M*, and *HPRT1*.

### PSA-ARE3-luciferase reporter assay

PC3 cells were seeded at a density of 5,000 cells per well in 96-well plates in phenol red–free RPMI media supplemented with 10% charcoal-stripped FBS and concurrently transfected with 0.5 μg/mL CONTROL-FLAG, AR-FL-FLAG, or AR-V7-FLAG and 0.25 μg/mL PSA-ARE3-luciferase, using X-tremeGENE HP DNA Transfection Reagent (Merck) as per the manufacturer’s recommendations and incubated overnight. The cells were then treated with vehicle (DMSO 0.1%), 250 nmol/L CCT365248, 250 nmol/L NXP800, or 3 μmol/L enzalutamide for 1 hour prior to either stimulation with 10 nmol/L dihydrotestosterone (DHT) or DHT vehicle (ethanol 0.1%). Cells were then incubated for 16 hours and lysed with Pierce IP Lysis Buffer (Thermo Fisher Scientific). Luciferase Assay Reagent (Promega) was added to lysate, and luciferase activity was measured using the BioTek Cytation 5 Cell Imaging Multimode Reader (Agilent).

### AR chromatin IP

22Rv1 cells were plated in hormone-deficient media for 72 hours. After which, cells were treated with CCT365248 (250 nmol/L) or NXP800 (250 nmol/L) for 1 hour, followed by stimulation with 10 nmol/L DHT or unstimulated with DHT vehicle (ethanol 0.1%) for 5 hours. Chromatin was extracted using the Chromatin Extraction Kit (Abcam), and chromatin IP (ChIP) was performed using the ChIP Kit Magnetic–One Step (Abcam) as per manufacturer’s recommendations. Briefly, cells were cross-linked with 1% fresh formaldehyde for 10 minutes at room temperature. Chromatin was sheared to 200 to 700 bp using Diagenode ultrasonicator for 30 cycles. Lysates were incubated with AR antibody (Millipore, Cat. #06-680, RRID: AB_310214). ChIP DNA was extracted with the aforementioned kit, and qPCR was performed using PowerUP SYBR (Thermo Fisher Scientific). Genes selected for ChIP-qPCR were known AR regulated genes: *KLK2*, *KLK3*, *FKBP5*, *TMPRSS2*, *CHRNA2*, and *ANKRD30B* (53). Primers were designed for AR binding sites and are listed in Supplementary Table S6. Results were expressed as mean binding as percentage of input with SD from two biological experiments with three replicates.

### *In vitro* patient-derived xenograft organoid proliferation

The development of CP50, CP89, and CP142 patient-derived xenografts (PDX) have been previously described (44, 52, 54, 55). CP129 was developed from the same patient as CP89 but from a biopsy taken later in the treatment timeline. PDX organoid (PDX-O) were derived from PDX and PDX-O proliferation studies performed as previously described (44). CellTiter-Glo 3D Luminescent Cell Viability Assay (Promega) was used to assay growth of PDX-O according to the manufacturer’s instructions, and luminescence was measured using Synergy HTX (BioTek).

### RNA sequencing and analyses (cell lines and castration-resistant VCaP prostate cancer cell line–derived mouse xenograft)

Prostate cancer cell line and castration-resistant VCaP prostate cancer cell line–derived mouse xenograft RNA quality was analyzed using the Agilent TapeStation RNA ScreenTape. About 500 ng of total RNA from each sample was first used in the NEBNext rRNA Depletion Kit, followed by the NEBNext Ultra II Directional RNA Library Prep Kit, according to the manufacturer’s instructions. Library quality was confirmed using the Agilent TapeStation High Sensitivity DNA ScreenTape. The libraries were quantified and normalized by qPCR using the KAPA Library Quantification Kit (Roche). Library clustering was performed on a cBot with Illumina HiSeq PE Cluster Kit v3. The libraries were sequenced as paired-end 101 bp reads on an Illumina HiSeq 2500 with an Illumina HiSeq SBS Kit v3. Base calling and quality scoring were performed using Real-Time Analyses (version 1.18.64) and FASTQ file generation and demultiplexing using CASAVA (RRID: SCR_001802). All FASTQ files were trimmed of adapter sequences using Trimmomatic (v0.39; RRID: SCR_011848). For prostate cancer cell line models (VCaP, LNCaP95, and 22Rv1, and newly developed 22Rv1 resistant sublines), paired-end reads were aligned to the human reference genome (GRCh38/hg38) using the STAR aligner (v2.7.7a; RRID: SCR_004463). For castration-resistant VCaP prostate cancer cell line–derived mouse xenograft, paired-end reads were aligned both to the human reference genome (GRCh38/hg38) and the mouse reference genome (GRCm38/mm10) using STAR. Aligned reads from both genomes were then used with XenofilteR (v1.6) to select only high-confidence human alignments. Subsequent to alignment, raw gene expression counts were computed using the Subread package (v2.0.1; RRID: SCR_009803) and used for differential expression analysis with the DESeq2 package (v1.32.0; RRID: SCR_015687). Normalized gene expression values were reported as calculated using DESeq2, using normalization to the geometric mean across replicates. Normalized counts were obtained for each model and experiment individually. The log fold change was obtained from DESeq2 with the parameters pAdjustMethod set to “false discovery rate” (FDR) and with independent filtering switched off. The differentially expressed genes were filtered for significance (adjusted *P* < 0.05), and the respective resulting lists of differentially expressed genes were used for pathway analysis with the GSEAPreranked algorithm from the clusterProfiler package (v4.0.5; RRID: SCR_016884) with default parameters. The gene set enrichment analysis was performed using gene sets comprising the H (Hallmark) collection from the MSigDB v7.1 (51). All normalized enrichment scores (NES) and FDRs presented for pathway analysis show the changes induced by NXP800 as compared with the closely matched inactive chemical control analog CCT365248–treated models or between an NXP800-resistant 22Rv1 prostate cancer cell subline and the corresponding controls or between control and heat shock.

### Development of a NXP800-resistant 22Rv1 prostate cancer subline

To develop a resistant subline, 22Rv1 cells were treated with increasing concentrations of NXP800. The 22Rv1 NXP800 subline (NXP800-R) was defined by the ability to maintain growth in the presence of 2.5 μmol/L NXP800. Control cell lines were developed by exposing 22Rv1 cells to the inactive, matched chemical control compound CCT365248 (inactive-C line) or 0.1% DMSO vehicle (vehicle-C line).

### *In vivo* castration-resistant VCaP prostate cancer cell line–derived mouse xenograft studies

VCaP prostate cancer cell line–derived mouse xenografts were established by subcutaneous injection of VCaP prostate cancer cells into the flank of uncastrated male nonobese diabetic SCID gamma (NSG) mice. Mice were then castrated 10 to 12 days prior to treatment commencement. Once tumors had established castration-resistant growth, animals were randomized into vehicle (10% DMSO/90% hydroxypropyl-β-cyclodextrin/citrate buffer, pH 5) or 35 mg/kg NXP800 (in vehicle) one-time-daily treatment groups. For short-term studies, mice were treated for 5 days by oral gavage, after which tumors were harvested for pharmacodynamic analyses. For long-term treatment, mice were treated for 38 days, by oral gavage. In all studies, the tumor volume (measured by caliper), animal body weight, and conditions were monitored at least two times weekly.

### Immunohistochemistry 

All tissue blocks were freshly sectioned and considered for IHC analyses of cleaved caspase 3 (Cell Signaling Technology, Cat. #9661, RRID: AB_2341188) or Ki-67 (Agilent, Cat. #M7240, RRID: AB_2142367). Cleaved caspase 3 and Ki-67 IHC was carried out as previously reported (18). Ki-67 and cleaved caspase 3 were reported as a percentage of cells that were positive for protein expression determined by a pathologist (B. Gurel) blinded to clinical data.

### Statistical analyses

Bioinformatic analyses are detailed in associated sections. For the GO cellular response to heat gene expression signature clinical analyses, patients were divided into two groups (≤80th vs. >80th percentile). OS was defined as the time from CRPC biopsy to date of death or last follow-up (censored). OS was estimated using the Kaplan–Meier method and log-rank test for significance. Hazard ratio (HR) with 95% confidence intervals (CI) and *P* values for univariate Cox survival models are shown. The unpaired Student *t* tests were used to determine significant differences between independent groups in all the following analyses. For prostate cancer cell lines and PDX-Os, the difference in growth, and the difference in RNA expression, subsequent to drug treatments was reported. Furthermore, the difference in PSA-ARE3-luciferase stimulation by various AR constructs in response to DHT after drug treatments was calculated, as well as AR DNA binding in response to DHT after drug treatments. Finally, the difference in growth, RNA, and protein expression in castration-resistant VCaP prostate cancer cell line–derived mouse xenograft studies after drug treatments was reported. Statistical analyses were performed using GraphPad Prism v7 (GraphPad Software; RRID: SCR_002798). All experimental replicates and statistical analyses performed are detailed in figure legends. Statistical significance was prespecified at *P* ≤ 0.05. No adjustment for multiple testing has been made.

### Study approvals

All clinical CRPC biopsy transcriptome analyses were performed on already published cohorts (2, 3, 48). All PDX models providing PDX-O were derived from patients who were treated at The Royal Marsden Hospital, had provided written informed consent, and were enrolled in institutional protocols approved by the ethics review committee. All mouse work was carried out in accordance with The ICR guidelines, including approval by The ICR Animal Welfare and Ethical Review Body, in compliance also with the UK Animals (Scientific Procedures) Act 1986 and the National Cancer Research Institute guidelines for the welfare and use of animals in cancer research (56).

### Data availability

The cell line and castration-resistant VCaP prostate cancer cell line–derived mouse xenograft RNA sequencing (RNA-seq) data are available under accession number PRJEB81176 from The European Nucleotide Archive. In addition to publicly deposited data, all data are contained within presented figures, and any further reasonable data access requests can be submitted to the corresponding authors.

## Results

### AR and AR-V7 bind members of the 70-kDa HSP family, and heat shock–mediated cellular stress increases HSP72 and AR-V7 protein expression in prostate cancer cells

HSPs have been shown to bind and activate both AR and AR-V7 in prostate cancer (17–28). To determine those proteins (including HSPs) that bind both AR and AR-V7, we conducted FLAG-tagged immunoprecipitation–mass spectrometry (IP-MS) on AR-negative PC3 prostate cancer cells overexpressing FLAG-tagged AR-FL and AR-V7 proteins (Supplementary Fig. S2A). Consistent with their described role in AR signaling, members of the 70-kDa HSP family are among the most prominent binding partners of both AR-FL and AR-V7 (Supplementary Fig. S2B; refs. 17, 19, 27, 28).

Because HSPs play a key role in cellular responses to stress, we next interrogated the impact of different cellular stresses (AR targeting, heat shock, and radiation) on AR-FL and AR-V7 protein expression in VCaP prostate cancer cells (Supplementary Fig. S2C). Consistent with previous reports, AR targeting with enzalutamide led to an increase in AR-V7 protein expression (Supplementary Fig. S2C; ref. 57). Interestingly, heat shock, with associated induction of HSP72 protein expression, was as effective as enzalutamide therapy at inducing AR-V7 protein expression (Supplementary Fig. S2C). By contrast, radiotherapy, with associated phosphorylation of replication protein A, did not impact AR-V7 protein levels (Supplementary Fig. S2C). Taken together, these data indicate that 70-kDa HSPs not only bind AR-FL and AR-V7 but their physiological induction by heat shock also associates with increased AR-V7 protein expression. This supports further investigation of targeting cellular response to heat stress components as a therapeutic strategy to overcome persistent AR signaling in lethal prostate cancer.

### GO cellular response to the heat gene expression signature associates with AR signaling and identifies poorer prognosis of metastatic CRPC in whole-biopsy RNA-seq analyses

**Figure 1.**
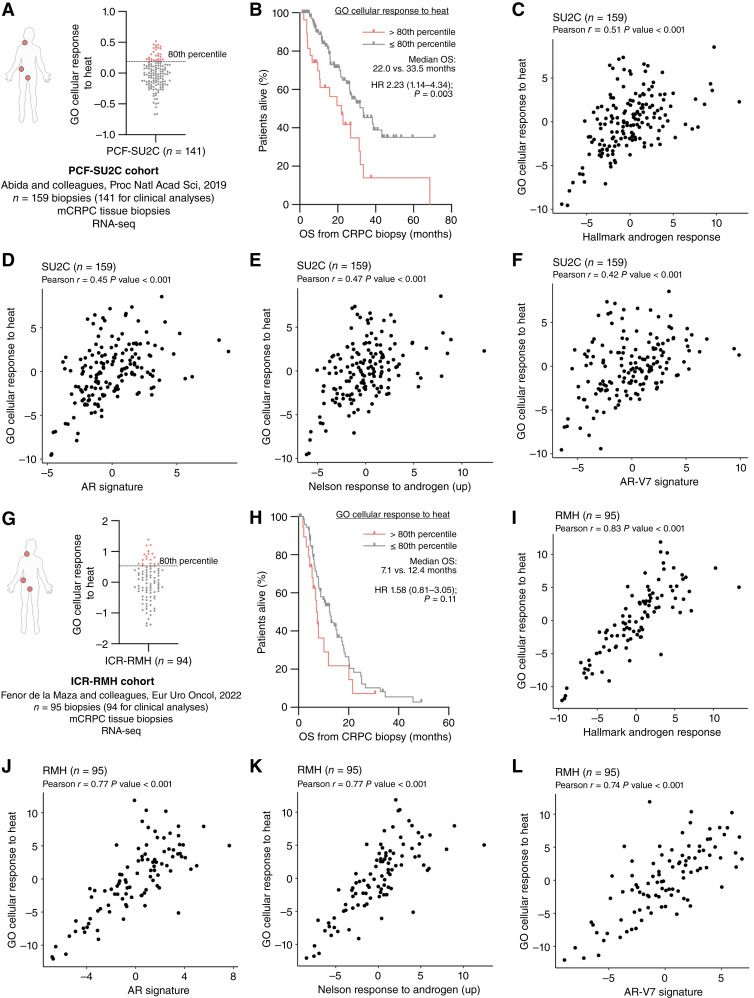
GO cellular response to heat gene expression signature associates with AR signaling and poorer prognosis in men suffering from CRPC. **A** and **G,** Two independent (PCF-SU2C and ICR-RMH) transcriptome cohorts of patients with CRPC. Quantification of GO cellular response to heat gene expression signature in each transcriptome cohort of patients with CRPC in the PCF-SU2C (**A**) and ICR-RMH (**G**) CRPC cohorts. Biopsies (red dots) with GO cellular response to heat gene expression signature >80th percentile (dotted line) are shown. **B** and **H,** Kaplan–Meier curves for OS from CRPC biopsy by >80th percentile (red) or ≤80th percentile (gray) GO cellular response to heat gene expression signature in PCF-SU2C (**B**) and ICR-RMH (**H**) transcriptome cohorts. Median OS is shown. HR with 95% CI and *P* values for univariate Cox survival model are shown. **C–F** and **I–L,** Association between GO cellular response to heat gene expression signature and Hallmark Androgen Response, AR signature, Nelson Response to Androgen Up, and AR-V7 signature in transcriptome cohorts of patients with PCF-SU2C (**C–F**) and ICR-RMH (**I–L**). Pearson *r* and *P* values are shown.

To further interrogate the clinical significance of these preclinical observations, we tested the association between GO cellular response to heat gene expression signature (GO:0034605; Supplementary Table S3) and OS in the two CRPC transcriptome cohorts (PCF-SU2C, *n* = 141; ICR-RMH, *n* = 94, Fig. 1A, B, G, and H; refs. 2, 48). The GO cellular response to heat gene expression signature was chosen on the basis of being the most significantly enriched MSigDB pathway matching the search term “heat” in VCaP prostate cancer cells after heat shock (Supplementary Fig. S2D). Patients with a higher (>80th percentile) GO cellular response to heat gene expression signature had poorer prognosis in both the PCF-SU2C [median OS 22.0 vs. 33.5 months, HR 2.23 (95% CI 1.14-4.34), *P* = 0.003] and ICR-RMH [median OS 7.1 vs. 12.4 months, HR 1.58 (0.81–3.05), *P* = 0.11] cohorts when compared with those patients with a lower (≤80th percentile) GO cellular response to heat gene expression signature (Fig. 1A, B, G, and H). Next, we determined whether the GO cellular response to heat gene expression signature was associated with previously reported AR signatures in three independent cohorts of patients with CRPC in transcriptome analysis (PCF-SU2C, *n* = 159; ICR-RMH, *n* = 95; UW, *n* = 185, Fig. 1A and G; Supplementary Fig. S3A; refs. 2, 3, 14, 48, 51, 52). The GO cellular response to heat gene expression signature significantly associated with the Hallmark Androgen Response (*r* = 0.51, *P* < 0.001), AR signature (*r* = 0.45, *P* < 0.001), Nelson Response to Androgen Up (*r* = 0.47, *P* < 0.001), and AR-V7 signature (*r* = 0.42, *P* < 0.001) in the PCF-SU2C cohort (Fig. 1C–F). Consistent with this, the GO cellular response to heat gene expression signature significantly associated with the Hallmark Androgen Response (*r* = 0.83, *P* < 0.001), AR signature (*r* = 0.77, *P* < 0.001), Nelson Response to Androgen Up (*r* = 0.77, *P* < 0.001), and AR-V7 signature (*r* = 0.74, *P* < 0.001) in the ICR-RMH cohort (Fig. 1I–L). In addition, in the UW cohort, the GO cellular response to heat gene expression signature significantly associated with the Hallmark Androgen Response (*r* = 0.39, *P* < 0.001), AR signature (*r* = 0.39, *P* < 0.001), Nelson Response to Androgen Up (*r* = 0.29, *P* < 0.001), and AR-V7 signature (*r* = 0.4, *P* < 0.001; Supplementary Fig. S3B–S3E). Taken together, these data demonstrate that the GO cellular response to heat gene expression signature associates with oncogenic AR signaling and identifies patients with advanced prostate cancer with poorer prognosis, further supporting the targeting of cellular response to heat stress as a therapeutic strategy in lethal prostate cancer.

### NXP800 inhibits AR transactivation and signaling to suppress the growth of AR and AR-V7 expressing castration-resistant prostate cancer models

Given the role that HSPs play in AR activity, and the demonstration that the GO Cellular Response to Heat gene expression signature associates with oncogenic AR signaling and poorer prognosis in advanced prostate cancer, we next explored whether targeting the HSF1 pathway, which regulates the canonical heat shock stress response and has been shown to modulate the AR, could represent a potential therapeutic strategy for the treatment of CRPC (18, 20, 28, 39, 58–61). To this end, we used NXP800, a clinical stage HSF1 pathway inhibitor that we derived from a phenotypic screen to detect inhibitors of the HSF1-mediated stress pathway (40, 43). Consistent with our published results in human ovarian cancer cells (43), NXP800 (Supplementary Fig. S1A), but not its closely matched inactive chemical control compound CCT365248 (Supplementary Fig. S1B), inhibited the induction of HSP72 protein expression in response to HSF1 activation, mediated by HSP90 inhibition, in a concentration-dependent manner in VCaP, LNCaP95 and 22Rv1 prostate cancer cells (Supplementary Fig. S4A–S4C). In addition, NXP800 reduced both basal, and HSP90 inhibitor-induced, HSP72 protein expression levels, in contrast to its inactive chemical control CCT365248, confirming its important effect in blocking HSF1-activated HSP expression in prostate cancer cells (Supplementary Fig. S4D).

**Figure 2.**
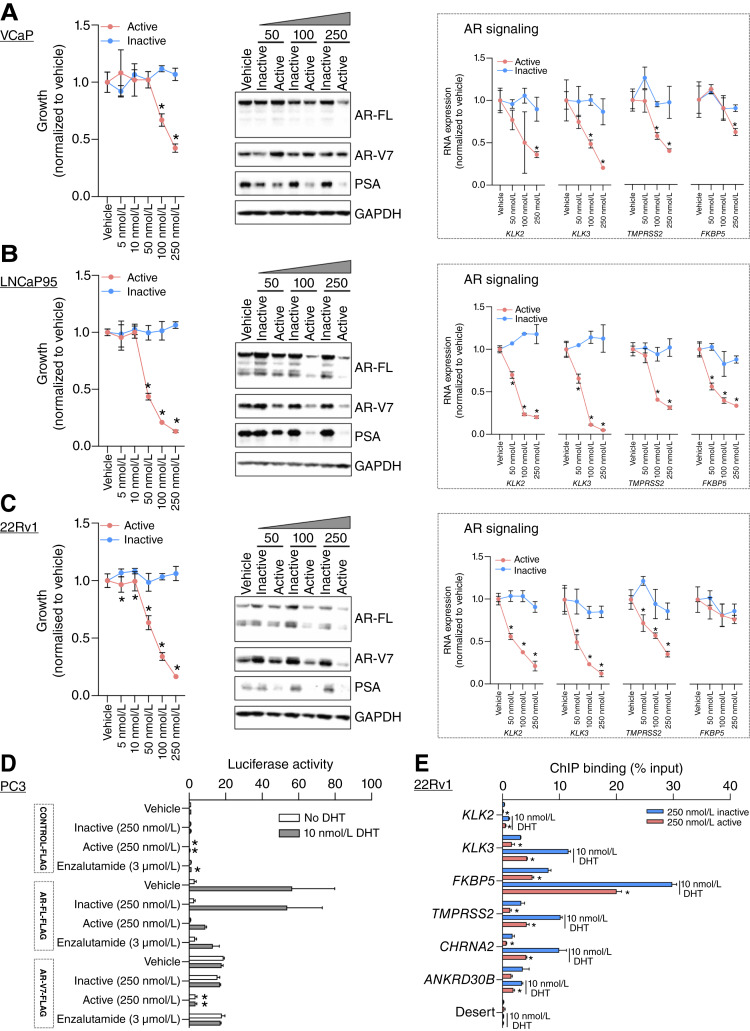
NXP800 inhibits AR transactivation and AR signaling to inhibit the growth of AR aberrant prostate cancer models. **A–C,** VCaP (**A**), LNCaP95 (**B**), and 22Rv1 (**C**) prostate cancer cells were treated with vehicle (DMSO 0.1%) or various concentrations (5, 10, 50, 100, and 250 nmol/L) of NXP800 (active, red line) or CCT365248 (inactive, blue line), and growth was determined after 5 days by CellTiter-Glo Luminescent Cell Viability Assay. Mean growth (compared with vehicle; defined as 1) with SD from a single experiment with six replicates is shown. *P* values were calculated for active compound compared with inactive compound for each concentration using the unpaired Student *t* test. *P* values ≤ 0.05 are shown (*). VCaP (**A**), LNCaP95 (**B**), and 22Rv1 (**C**) prostate cancer cells were treated with vehicle (DMSO 0.1%) or various concentrations (50, 100, and 250 nmol/L) of NXP800 (active) or CCT365248 (inactive) for 48 hours, and AR-FL, AR-V7, PSA, and GAPDH protein expression was determined from one experiment performed in triplicate. Single Western blot representative of three is shown. VCaP (**A**), LNCaP95 (**B**), and 22Rv1 (**C**) prostate cancer cells were treated with vehicle (DMSO 0.1%) or various concentrations (50, 100, and 250 nmol/L) of NXP800 (active, red line), or CCT365248 (inactive, blue line) for 48 hours, and *KLK2*, *KLK3*, *TMPRSS2*, and *FKBP5* RNA expression was determined. Mean RNA expression (normalized to average of *GAPDH/B2M/HRPT1/RPLP0* and vehicle; defined as 1), with SD from a single experiment with three replicates is shown. *P* values were calculated for active compared with inactive compound for each concentration using the unpaired Student *t* test. *P* values ≤ 0.05 are shown (*). **D,** PC3 cells were transfected with CONTROL-FLAG, AR-FL-FLAG, or AR-V7-FLAG and PSA-ARE3-luciferase, prior to treatment with vehicle (DMSO 0.1%), 250 nmol/L CCT365248 (inactive), 250 nmol/L NXP800 (active), or 3 μmol/L enzalutamide for 1 hour prior to stimulation with or without 10 nmol/L DHT for 16 hours. Mean luciferase activity (compared with CONTROL-FLAG/vehicle/without DHT) with SD from two experiments with five replicates is shown. *P* values were calculated for each plasmid (with and without DHT stimulation) with vehicle compared with other treatments using the unpaired Student *t* test. *P* values ≤ 0.05 are shown (*). **E,** ChIP with an AR antibody was carried out in 22Rv1 cells that were plated in starved (charcoal-striped serum/phenol red–free) media for 72 hours prior to treatment with 250 nmol/L CCT365248 (inactive) or 250 nmol/L NXP800 (active) for 1 hour prior to stimulation with or without 10 nmol/L DHT for 5 hours. AR recruitment to AR-responsive genes (*KLK2*, *KLK3*, *FKBP5*, *TMPRSS2*, *CHRNA2*, and *ANKRD30B*) was determined. Mean binding as percentage of input with SD from two experiments with three replicates is shown. *P* values were calculated for 250 nmol/L CCT365248 (inactive) compared with 250 nmol/L NXP800 (active) with and without DHT stimulation using the unpaired Student *t* test. *P* values ≤ 0.05 are shown (*).

Next, we determined the impact of NXP800 on the growth of both AR signaling inhibitor (ARSI)-sensitive (VCaP) and ARSI-resistant (LNCaP95 and 22Rv1) prostate cancer cell lines. Five days of treatment with NXP800, but not its inactive chemical control CCT365248, significantly inhibited the growth of all three AR-expressing cell lines, including LNCaP95 and 22Rv1 that also express AR-V7, and are resistant to enzalutamide, suggesting that NXP800 treatment may overcome endocrine resistance (Fig. 2A–C). Considering the link between HSPs and AR activity, we next investigated the impact of NXP800 on AR signaling, demonstrating a decrease in AR-FL and AR-V7 protein expression, and also in expression of AR-responsive genes (*KLK2*, *KLK3*, *TMPRSS2*, and *FKBP5*), across all three prostate cancer cell lines (Fig. 2A–C). Having demonstrated that NXP800, but not its inactive control CCT365248, suppressed AR-responsive genes across multiple prostate cancer cell lines, we further investigated the impact of NXP800 on AR biology. Thus, we determined the impact of NXP800, its inactive chemical control CCT365248, and also enzalutamide, on transfected AR-FL or AR-V7 transactivation of a PSA-ARE3-driven luciferase reporter assay system in AR negative PC3 prostate cancer cells (Fig. 2D). Both NXP800 and enzalutamide, but not the inactive chemical control for NXP800, CCT365248, inhibited DHT-mediated AR-FL transactivation (Fig. 2D). In addition, unlike its inactive chemical control CCT365248 and enzalutamide, NXP800 inhibited AR-V7 transactivation (Fig. 2D). These data support previous reports that HSPs are critical for both AR-FL and AR-V7 activity (13, 14, 18, 20, 28). Next, using chromatin-immunoprecipitation technology with an AR antibody pull-down in 22Rv1 cells, we demonstrated that NXP800, but not its inactive chemical control CCT365248, reduced AR binding at multiple AR-responsive genes including *KLK2*, *KLK3*, *FKBP5*, *TMPRSS2*, *CHRNA2*, and *ANKRD30B* (Fig. 2E; ref. 53). These data are in contrast to enzalutamide, which inhibits AR signaling in enzalutamide-responsive VCaP cells but has little impact on AR signaling in enzalutamide-resistant LNCaP95 and 22Rv1 cells when compared with NXP800 (Fig. 2A–C; Supplementary Fig. S5A–S5C).

**Figure 3.**
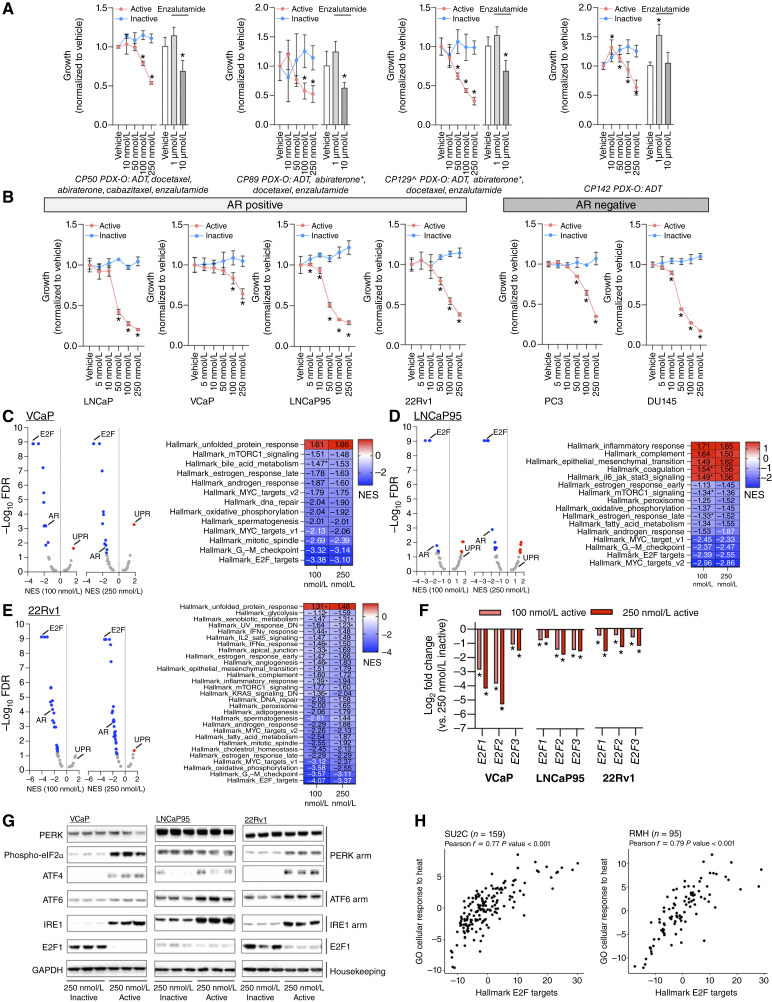
NXP800 inhibits the growth of AR-dependent and AR-independent prostate cancer models with activation of the UPR and inhibition of key signaling pathways. **A** and **B,** PDX-O [CP50, CP89, CP129, and CP142 (**A**)], AR-positive (VCaP, LNCaP, LNCaP95, and 22Rv1), and AR-negative (PC3 and DU145) prostate cancer cell lines (**B**) were treated with vehicle (DMSO 0.1%) or various concentrations (5, 10, 50, 100, and 250 nmol/L) of NXP800 (active, red line), CCT365248 (inactive, blue line), and in the case of organoids, various concentrations (1 and 10 μmol/L) of enzalutamide (gray scale), and growth was determined after 5 days for cell lines and 7 days for organoids by CellTiter-Glo Cell Viability Assay as defined in “Materials and Methods.” Mean growth (compared with vehicle; defined as 1) with SD from a single experiment with three to six replicates is shown. *Abiraterone given in the castration-sensitive setting. ^CP89 and CP129 derived from two temporally separated mCRPC biopsies from the same patient. *P* values were calculated for NXP800 compared with CCT365248 for each concentration and for enzalutamide, compared with vehicle using the unpaired Student *t* test. *P* values ≤ 0.05 are shown (*). **C–E,** VCaP, LNCaP95, and 22Rv1 prostate cancer cells were treated with NXP800 (active, 100 or 250 nmol/L) or CCT365248 (inactive, 250 nmol/L) for 48 hours. RNA-seq was performed on each single experiment in triplicate (duplicate for 250 nmol/L NXP800 in LNCaP95). Analysis of RNA-seq with gene set enrichment analysis shows the enrichment and de-enrichment of Hallmark pathways in response to 100 and 250 nmol/L NXP800 (compared with 250 nmol/L CCT365248) in VCaP (**C**), LNCaP95 (**D**), and 22Rv1 (**E**) prostate cancer cells. NES and FDR are shown as volcano plots. Colored dots denote significantly (FDR 0.05) enriched (red dots) and de-enriched (blue dots) pathways with NXP800 (active compound) treatment. Table shows the NES associated with pathways wherein the FDR was ≤0.05 for 100 and/or 250 nmol/L (asterisk indicates those in which FDR was >0.05). **F,** VCaP (**A**), LNCaP95 (**B**), and 22Rv1 (**C**) prostate cancer cells were treated with NXP800 (active, 100 or 250 nmol/L) or CCT365248 (inactive, 250 nmol/L) for 48 hours. RNA-seq was performed on each single experiment in triplicate (duplicate for 250 nmol/L NXP800 in LNCaP95). Log_2_ fold expression level changes of “activating” *E2F* (*E2F1*–*3*) family members treated with NXP800 (active, 100 or 250 nmol/L) were compared with CCT365248 (inactive, 250 nmol/L). *P* values were calculated by DESeq2 using the Wald test. *P* values ≤ 0.05 are shown (*). **G,** VCaP, LNCaP95, and 22Rv1 prostate cancer cells were treated with 250 nmol/L CCT365248 (inactive) or 250 nmol/L NXP800 (active) for 24 hours. PERK, phospho-eIF2α, and ATF4 (PERK arm); ATF6 (ATF6 arm); IRE1 (IRE1 arm); E2F1 (E2F); and GAPDH (housekeeping) protein expression was determined by Western blot from one experiment performed in triplicate. **H,** Association between GO cellular response to heat gene expression signature and Hallmark E2F Targets in transcriptome cohorts of patients with PCF-SU2C and ICR-RMH. Pearson *r* and *P* values are shown.

Next, to further explore the utility of NXP800 in CRPC, we evaluated treatment in four PDX-Os derived from biopsies of four patients with metastatic CRPC (from three separate patients; Fig. 3A; refs. 44, 52, 54, 55). NXP800, but not the inactive chemical control CCT365248, inhibited growth in all models studied (Fig. 3A). In contrast, AR-targeting with enzalutamide demonstrated varying responses across models, with growth inhibition only seen at the highest concentrations explored (Fig. 3A). Taken together, these data demonstrate that NXP800 abrogates AR signaling, including AR-V7 transactivation, with associated growth reduction in ARSI-resistant prostate cancer cell lines and patient-derived prostate cancer models, suggesting that it may provide an attractive strategy to overcome persistent AR signaling and treatment resistance in lethal prostate cancer.

### NXP800 suppresses AR-dependent and AR-independent prostate cancer model growth with activation of the UPR and inhibition of the key signaling pathways required for the development and progression of CRPC

To investigate whether the growth inhibitory effects of NXP800 were exclusively through AR signaling abrogation, we interrogated AR-positive (VCaP, LNCaP, LNCaP95, and 22Rv1) and AR-negative (PC3 and DU145) prostate cancer cell lines. NXP800, but not the inactive chemical control CCT365248, significantly inhibited the growth of all prostate cancer cell lines, independent of their AR status, suggesting that growth inhibition did not occur through AR signaling inhibition alone (Fig. 3B).

Having demonstrated that NXP800 has growth inhibitory effects in AR-dependent and AR-independent prostate cancer models, we next investigated further the effects of the drug by performing RNA-seq on VCaP, LNCaP95, and 22Rv1 prostate cancer cells subsequent to NXP800 treatment (Fig. 3C–E; Supplementary Tables S7–S9). Consistent with our targeted AR studies, 100 and 250 nmol/L NXP800 (in contrast to its inactive chemical control, CCT365248, at 250 nmol/L) substantially impacted AR signaling with de-enrichment of the Hallmark Androgen Response in VCaP (100 nmol/L, NES −1.87, FDR <0.001; 250 nmol/L, NES −1.60, FDR 0.0134), LNCaP95 (100 nmol/L, NES −1.53, FDR 0.0165; 250 nmol/L, NES −1.87, FDR 0.0012), and 22Rv1 (100 nmol/L, NES −2.29, FDR <0.001; 250 nmol/L, NES −1.88, FDR <0.001) prostate cancer cells (Fig. 3C–E; Supplementary Tables S7–S9). Interestingly, exposure to NXP800 (in contrast to its inactive control compound, CCT365248, at 250 nmol/L) also led to activation of the UPR in prostate cancer cells (Fig. 3C–E; Supplementary Tables S7–S9). Thus, treatment with 100 and 250 nmol/L NXP800 (but not its inactive control compound, CCT365248, at 250 nmol/L) led to the enrichment of the Hallmark UPR in VCaP (100 nmol/L, NES 1.61, FDR 0.0232; 250 nmol/L, NES 1.86, FDR <0.001) and 22Rv1 (100 nmol/L, NES 1.31, FDR 0.1276; 250 nmol/L, NES 1.48, FDR 0.0373), but not significantly in LNCaP95 (100 nmol/L, NES 1.38, FDR 0.0941; 250 nmol/L, NES 1.18, FDR 0.2443) prostate cancer cells (Fig. 3C–E; Supplementary Tables S7–S9). In addition, other signaling pathways including Hallmark MYC Targets V1 and V2, Hallmark E2F Targets and Hallmark G_2_–M Checkpoint, which are all implicated in the development and progression of CRPC, were consistently de-enriched in response to NXP800 treatment (Fig. 3C–E; Supplementary Tables S7–S9).

Next, having identified through our RNA-seq analyses that treatment with NXP800 increased the UPR gene expression signature and decreased E2F targets, these effects were investigated further (Fig. 3F and G). Given the decrease in E2F targets, we further interrogated the expression of “activating” *E2Fs* within our RNA-seq analyses, demonstrating that *E2F1* to *E2F3* RNA expression was significantly decreased in response to NXP800 treatment across all prostate cancer cells studied (Fig. 3F; refs. 62, 63). In addition, orthogonal validation by Western blot analysis demonstrated that E2F1 protein expression was reduced in response to NXP800 treatment across all prostate cancer cells studied (Fig. 3G). Furthermore, the GO cellular response to heat gene expression signature that is associated with oncogenic AR signaling and poorer prognosis in patients with advanced prostate cancer, also significantly associated with Hallmark E2F Targets in PCF-SU2C (*r* = 0.77, *P* < 0.001) and ICR-RMH (*r* = 0.79, *P* < 0.001) CRPC transcriptome cohorts (Fig. 3H; refs. 2, 48). Finally, treatment with 250 nmol/L NXP800, but not its inactive chemical control CCT365248 at this concentration, resulted in increased eIF2α phosphorylation and ATF4, ATF6, and IRE1 protein expression in VCaP, LNCaP95, and 22Rv1 prostate cancer cells (Fig. 3G).

Taken together, these data confirmed that treatment with NXP800 suppresses AR signaling and provided novel insights into the mechanism of action of NXP800 in prostate cancer cells, with activation of the UPR and inhibition of AR, MYC, and E2F activity—all reported to be key to the development and progression of CRPC (5, 64–66).

### Activation of the UPR may impact NXP800-mediated abrogation of AR and E2F function in prostate cancer models

**Figure 4.**
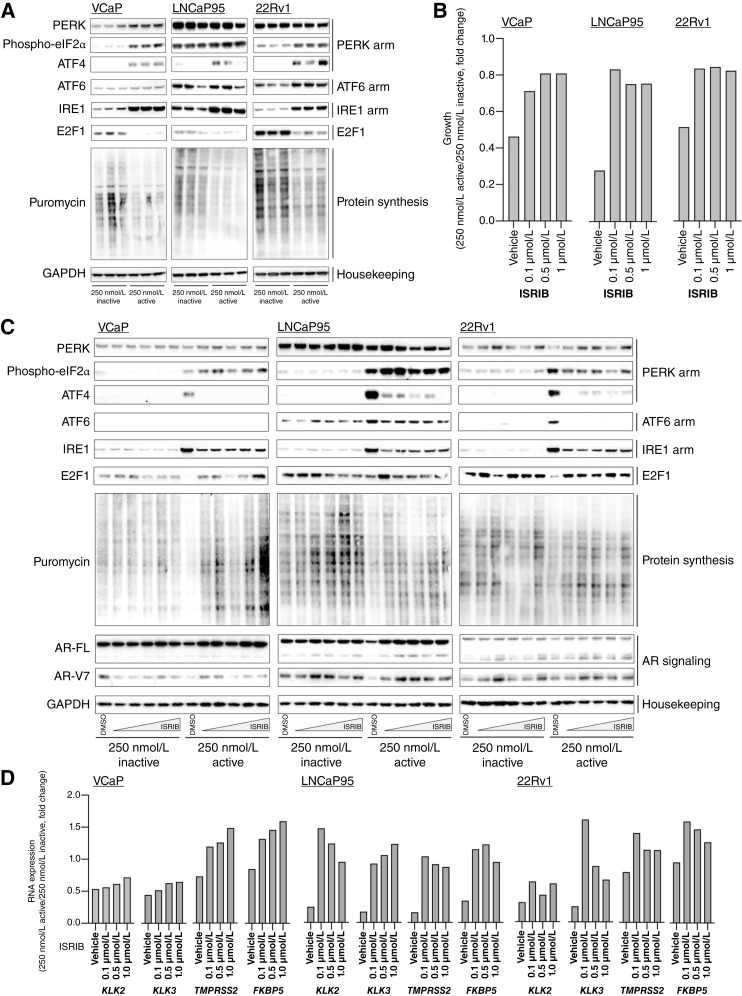
NXP800 activates the UPR and inhibits key signaling pathways identifying a novel mechanism of action in prostate cancer models. **A,** VCaP, LNCaP95, and 22Rv1 prostate cancer cells were treated with 250 nmol/L CCT365248 (inactive) or 250 nmol/L NXP800 (active) for 24 hours prior to the addition of puromycin for 30 minutes. PERK, phospho-eIF2α, and ATF4 (PERK arm); ATF6 (ATF6 arm); IRE1 (IRE1 arm) protein; E2F1; puromycin (incorporation a surrogate for protein synthesis); and GAPDH (housekeeping) protein expression was determined by Western blot from one experiment performed in triplicate. **B,** VCaP, LNCaP95, and 22Rv1 prostate cancer cells were treated with 250 nmol/L NXP800 (active) or 250 nmol/L CCT365248 (inactive) with various concentrations of the small-molecule ISRIB (0.1, 0.5, and 1 μmol/L), and growth was determined after 5 days by CellTiter-Glo Luminescent Cell Viability Assay. Mean growth from a single experiment with four replicates was determined. Growth (fold change) between 250 nmol/L NXP800 and 250 nmol/L CCT365248 with various concentrations of ISRIB (0.1, 0.5, and 1 μmol/L) is shown. **C,** VCaP, LNCaP95, and 22Rv1 prostate cancer cells were treated with 250 nmol/L CCT365248 (inactive) or 250 nmol/L NXP800 (active) with various concentrations of ISRIB (0.1, 0.5, 1, 2, and 5 μmol/L) for 24 hours prior to the addition of puromycin for 30 minutes. PERK, phospho-eIF2α, and ATF4 (PERK arm); ATF6 (ATF6 arm); IRE1 (IRE1 arm); E2F1; puromycin (incorporation a surrogate for protein synthesis); AR-FL and AR-V7; and GAPDH (housekeeping) protein expression was determined by Western blot from one experiment. **D,** VCaP, LNCaP95, and 22Rv1 prostate cancer cells were treated with 250 nmol/L CCT365248 (inactive) or 250 nmol/L NXP800 (active) with various concentrations of ISRIB (0.1, 0.5, and 1 μmol/L) for 48 hours, and *KLK2*, *KLK3*, *TMPRSS2*, and *FKBP5* RNA expression was determined. Mean RNA expression (normalized to average of *GAPDH/B2M/HRPT1/RPLP0*) from a single experiment with three replicates is shown. RNA expression (fold change) between 250 nmol/L NXP800 and 250 nmol/L CCT365248 with various concentrations of ISRIB (0.1, 0.5, and 1 μmol/L) is shown.

Having determined that NXP800 activates the UPR gene signature and blocks AR and E2F activity in multiple prostate cancer models, we investigated whether, at least in part, these molecular mechanisms could be linked. Consistent with these discoveries, we further confirmed that treatment of VCaP, LNCaP95 and 22Rv1 prostate cancer cells with NXP800, but not the inactive chemical control CCT365248, increased eIF2α phosphorylation and ATF4, ATF6, and IRE1 protein expression. In addition, we demonstrated that treatment with NXP800 decreased incorporation of puromycin into nascent proteins, indicating reduced overall protein synthesis (Fig. 4A). To further explore whether the molecular mechanisms and phenotype described were a consequence of the activation of the UPR, we next determined whether the small-molecule ISRIB—which promotes the assembly, stabilization, and activation of the eukaryotic translation-initiation factor eIF2B decamer and allows protein synthesis to continue despite eIF2α phosphorylation subsequent to cell stress (67–69)—could rescue the observed NXP800-mediated phenotype (Fig. 4B–D; Supplementary Fig. S6).

The addition of ISRIB in VCaP, LNCaP95, and 22Rv1 prostate cancer cells resulted in a partial rescue of NXP800-mediated growth inhibition (Fig. 4B; Supplementary Fig. S6A). In addition, ISRIB partially reversed the impact of NXP800 on ATF4, ATF6, and IRE1 protein expression, decrease in protein expression of AR-FL/AR-V7 and E2F1, and inhibition of overall protein synthesis (Fig. 4C). Furthermore, ISRIB partially rescued NXP800-mediated suppression of AR-responsive genes (*KLK2*, *KLK3*, *TMPRSS2*, and *FKBP5*), whereas the ISRIB/CCT365248 combination had no effect on these targets (Fig. 4D; Supplementary Fig. S6B). Together with the gene expression data, our findings provide further evidence that the NXP800-mediated phenotype in prostate cancer models is, at least in part, driven by activation of the UPR.

### Mechanistic investigation of the NXP800-resistant 22Rv1 prostate cancer cell subline

**Figure 5.**
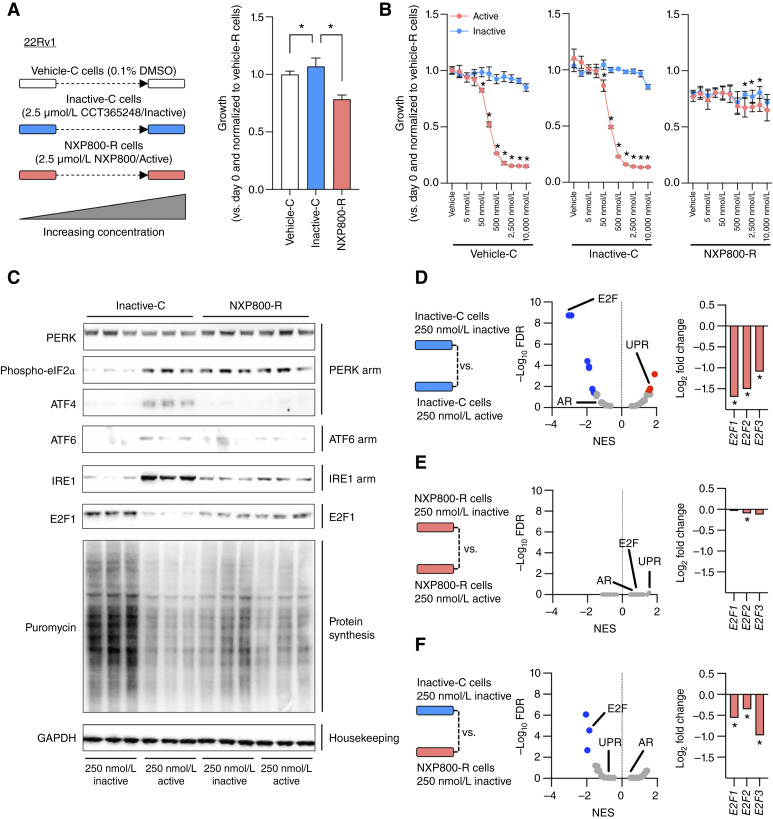
NXP800-resistant 22Rv1 prostate cancer cell sublines demonstrate the reversal of the NXP800-mediated phenotype. **A,** Long-term treatment of 22Rv1 prostate cancer cells with increasing concentrations (up to 2.5 μmol/L) of DMSO (vehicle-C, white), CCT365248 (inactive-C, blue), and NXP800 (NXP800-R, red) led to the generation of 22Rv1 prostate cancer cell–derived sublines. Mean growth was determined after 5 days by CellTiter-Glo Luminescent Cell Viability Assay compared with day 0 and vehicle-C cells with SD for each subline developed is shown. *P* values were calculated for subline compared with vehicle-C using the unpaired Student *t* test. *P* values ≤ 0.05 are shown (*). **B,** The impact of NXP800 and CCT365248 on the growth of vehicle-C, inactive-C, and NXP800-R sublines was determined. Mean growth in response to various concentrations of NXP800 (active, red line) and CCT365248 (inactive, blue line) was determined after 5 days by CellTiter-Glo Luminescent Cell Viability Assay compared with day 0 and vehicle-C cells with SD for each subline developed is shown. *P* values were calculated for active compared with inactive for each subline using the unpaired Student *t* test. *P* values ≤ 0.05 are shown (*). **C,** Inactive-C and NXP800-R sublines were treated with 250 nmol/L CCT365248 (inactive) or 250 nmol/L NXP800 (active) for 24 hours prior to the addition of puromycin for 30 minutes. PERK, phospho-eIF2α, and ATF4 (PERK arm); ATF6 (ATF6 arm); IRE1 (IRE1 arm); E2F1; puromycin (incorporation a surrogate for protein synthesis); and GAPDH (housekeeping) protein expression was determined by Western blot from one experiment performed in triplicate. **D–F**, Inactive-C and NXP800-R sublines were treated with either 250 nmol/L CCT365248 (inactive) or 250 nmol/L NXP800 (active) for 48 hours. RNA-seq was performed on each single experiment in triplicate. Analysis of RNA-seq with gene set enrichment analysis shows the enrichment and de-enrichment of Hallmark pathways comparing 250 nmol/L CCT365248 (inactive) vs. 250 nmol/L NXP800 (active) treated inactive-C sublines (**D**), 250 nmol/L CCT365248 (inactive) vs. 250 nmol/L NXP800 (active) treated NXP800-R sublines (**E**), and 250 nmol/L CCT365248 (inactive) treated inactive-C sublines vs. 250 nmol/L CCT365248 (inactive) treated NXP800-R sublines (**F**). NES and FDR are shown as volcano plots. Colored dots denote significantly (FDR 0.05) enriched (red dots) and de-enriched (blue dots) pathways. Log_2_ fold expression level changes of “activating” *E2F* (*E2F1*–*3*) family members comparing 250 nmol/L CCT365248 (inactive) vs. 250 nmol/L NXP800 (active) treated inactive-C sublines (**D**), 250 nmol/L CCT365248 (inactive) vs. 250 nmol/L NXP800 (active) treated NXP800-R sublines (**E**), and 250 nmol/L CCT365248 (inactive) treated inactive-C sublines vs. 250 nmol/L CCT365248 (inactive) treated NXP800-R sublines (**F**). P values were calculated by DESeq2 using the Wald test. P values ≤ 0.05 are shown (*).

To further evaluate the NXP800-mediated phenotype, we generated an NXP800 (NXP800-R)–resistant 22Rv1 prostate cancer subline derivative that maintained growth at high concentrations (2.5 μmol/L; Fig. 5A). Alongside the generation of NXP800-R, control cell lines were developed by exposing 22Rv1 cells to the inactive chemical control compound CCT365248 (inactive-C) or to 0.1% DMSO vehicle (vehicle-C). Characterization of these sublines demonstrated that the NXP800-R subline is indeed resistant to NXP800 treatment at high concentrations (up to 10 μmol/L) in contrast to both the vehicle-C and inactive-C sublines that respond to NXP800 in a concentration–responsive manner similar to the parental 22Rv1 cell line (Fig. 5B). In addition, in contrast to the vehicle-C and inactive-C control and parental cell 22Rv1 cell lines, NXP800 treatment had no effect on basal, or HSP90 inhibitor–induced, HSP72 protein expression in the NXP800-R subline (Supplementary Fig. S7).

Next, we further investigated the mechanism of action of NXP800 using our NXP800-R and inactive-C sublines. Consistent with our results in the 22Rv1 parental line, NXP800 treatment in the inactive-C subline led to increased eIF2α phosphorylation and ATF4, ATF6, and IRE1 protein expression, reduced protein synthesis, decreased AR signaling with reduced AR-FL/AR-V7 protein expression and AR DNA binding, and decreased E2F1 protein expression (Fig. 5C; Supplementary Fig. S8A–S8C). By contrast, treatment with NXP800 in the NXP800-R subline had limited/no effect on these molecular markers identified in both the NXP800-treated inactive-C and 22Rv1 parental lines (Fig. 5C; Supplementary Fig. S8A–S8C). In addition, the NXP800-R subline had a reduced basal growth rate with lower basal protein synthesis, AR signaling, and E2F1 protein expression, when compared with the inactive-C subline (Fig. 5A–C; Supplementary Fig. S8B).

Furthermore, when comparing RNA-seq analyses across these models, consistent with our previous studies described earlier, treating inactive-C sublines with 250 nmol/L NXP800 led to a significant enrichment of Hallmark UPR and de-enrichment of Hallmark E2F Targets with significantly lower “activating” *E2F* (*E2F1*–*3*) RNA levels when compared with treatment with 250 nmol/L of the inactive chemical control CCT365248 (Fig. 5D; Supplementary Table S10). By contrast, treatment of the NXP800-R subline with 250 nmol/L NXP800 did not show any changes in hallmark signaling pathways, and the RNA levels of “activating” *E2Fs* were minimally affected (Fig. 5E; Supplementary Table S11). Finally, only Hallmark E2F Targets and Hallmark MYC Targets V1 and V2 were significantly de-enriched when comparing inactive-C and NXP800-R sublines, with “activating” *E2F* (*E2F1*–*3*) RNA levels being significantly lower in the NXP800-R subline (Fig. 5F; Supplementary Table S12).

Taken together, these data support our earlier findings, confirming that NXP800 antiproliferative activity is mediated, in part, through the activation of the UPR with associated blockade of key signaling pathways implicated in CRPC progression.

### NXP800 demonstrates antitumor activity with associated activation of the UPR and suppression of E2F-mediated transcription in a castration-resistant VCaP prostate cancer cell line–derived mouse xenograft

**Figure 6.**
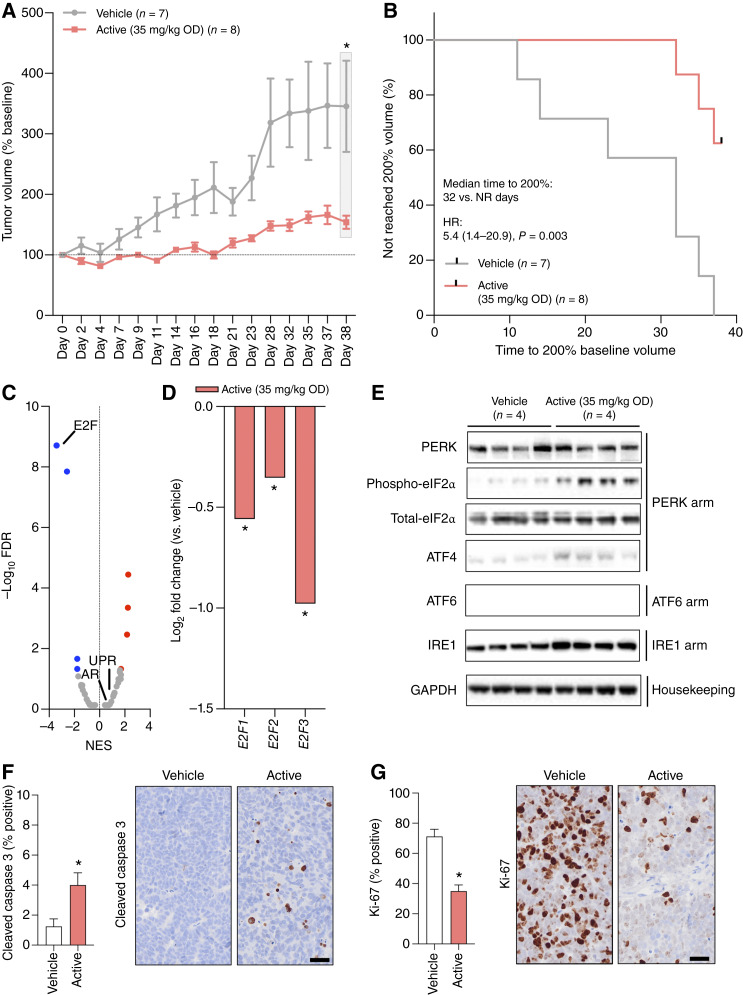
NXP800 activates the UPR and inhibits E2F-mediated transcription to drive antitumor activity against the castration-resistant VCaP prostate cancer cell line–derived mouse xenograft. **A** and **B,** Castration-resistant emergent VCaP prostate cancer cell line–derived mouse xenografts were treated with vehicle (*n* = 7, gray) or 35 mg/kg NXP800 (active, *n* = 8, red) after tumors had established castration-resistant growth with a defined dosing schedule for 38 days. Mean tumor volume (normalized to start; defined as 100%) with SEM is shown. *P* values were calculated for NXP800 compared with vehicle using the unpaired Student *t* test at 38 days. *P* values ≤ 0.05 are shown (*; **A**). Time to reach 200% starting tumor volume was used as a surrogate endpoint for survival. HR with 95% CI and *P* value for univariate Cox survival model are shown (**B**). **C–G,** Castration-resistant VCaP prostate cancer cell line–derived mouse xenografts were treated with vehicle (*n* = 4) or 35 mg/kg NXP800 (active, *n* = 4) daily after tumors had established castration-resistant growth for 5 days with tumor collection 6 hours after final dose for pharmacodynamic studies. RNA-seq was performed on tumor samples. Analysis of RNA-seq with gene set enrichment analysis shows the enrichment and de-enrichment of Hallmark pathways comparing NXP800 (active) and vehicle. NES and FDR are shown as volcano plots. Colored dots denote significantly (FDR 0.05) enriched (red dots) and de-enriched (blue dots) pathways (**C**). Log_2_ fold expression level changes of “activating” *E2F* (*E2F1*–*3*) family members treated with NXP800 (active) were compared with vehicle. *P* values were calculated by DESeq2 using the Wald test. *P* values ≤ 0.05 are shown (*; **D**). PERK, phospho-eIF2α, total-eIF2α, and ATF4 (PERK arm); ATF6 (ATF6 arm); IRE1 (IRE1 arm); and GAPDH (housekeeping) protein expression was determined by Western blot (**E**). Cleaved caspase 3 (**F**) and Ki-67 (**G**) protein expression was determined by IHC on formalin-fixed, paraffin-embedded tumors. Representative micrographs are shown. Scale bar, 50 μm. Mean percentage positive cells with SD are shown. *P* values were calculated for NXP800 (red) compared with vehicle (white) using the unpaired Student *t* test. *P* values ≤ 0.05 are shown (*).

Having demonstrated that NXP800 inhibits the growth of ARSI-resistant prostate cancer models by activating the UPR with associated suppression of key signaling pathways required for the development and progression of CRPC *in vitro*, we next evaluated the impact of the drug on a castration-resistant VCaP prostate cancer cell line–derived mouse xenograft. VCaP prostate cancer cell line–derived mouse xenografts *in vivo* that had developed castration resistance were treated orally with 35 mg/kg NXP800 or vehicle, either for 5 consecutive days (pharmacodynamic analyses) or over a 38-day period (efficacy analyses). The regimen used incorporated dose interruptions to allow for recovery from any weight loss due to NXP800 treatment, when compared with vehicle (Supplementary Fig. S9A–S9D). Importantly, NXP800 treatment significantly reduced VCaP prostate cancer cell line–derived mouse xenograft tumor growth in 38 days, also reducing the time for tumors to reach 200% volume when compared with the vehicle [median time to 200%: 32 days vs. not reached, HR 5.4 (95% CI 1.4–20.9), *P* = 0.003; Fig. 6A and B]. Evaluation of VCaP prostate cancer cell line–derived mouse xenograft tumors after 5 days of NXP800 treatment by RNA-seq demonstrated significant de-enrichment of Hallmark E2F Targets together with significantly decreased “activating” *E2F* (*E2F1*–*3*) RNA levels when compared with vehicle controls (Fig. 6C and D). Although these RNA-seq analyses did not demonstrate significant changes in the Hallmark Androgen Response or Hallmark UPR signatures, further orthogonal analyses did show increases in eIF2α phosphorylation, and ATF4 protein expression, but limited impact on AR-FL, AR-V7, and AR signaling (Fig. 6E; Supplementary Fig. S10A and S10B; Supplementary Table S13). Finally, consistent with the inhibition of xenograft tumor growth observed, NXP800 treatment led to increased cleaved caspase 3, indicating apoptosis, and reduced Ki-67, indicating decreased proliferation, when compared with the vehicle control (Fig. 6F and G; Supplementary Fig. S10C). Overall, these data demonstrate that NXP800 activates the UPR and suppresses key signaling pathways, including the E2Fs, to inhibit the growth of castration-resistant VCaP prostate cancer cell line–derived mouse xenografts *in vivo*, supporting its consideration for prostate cancer–specific clinical development.

## Discussion

Despite the development of multiple new treatments for advanced prostate cancer, treatment resistance in the clinic is inevitable and commonly driven by the reactivation of AR signaling (6–14). The development of innovative therapeutic approaches that suppress AR signaling through novel mechanisms of action remains an urgent unmet clinical need for prostate cancer medicine. One attractive strategy is to target AR co-regulators such as HSPs that have been shown to be critical for AR signaling in prostate cancer models (17–28). The studies herein provide further evidence to support the biological importance of HSPs in advanced prostate cancer, demonstrating that both AR and AR-V7 bind members of the 70-kDa HSP family. In addition, heat shock induction of HSP72 protein expression is as effective as enzalutamide treatment at increasing AR-V7 protein expression. These data in prostate cancer cells indicate that components of the molecular response to heat stress facilitate AR splicing, persistent AR signaling, and treatment resistance and help validate intervention in this pathway as a potential therapeutic approach. We further describe preclinical therapeutic and mechanistic studies to support the potential for the clinical-stage developmental HSF1 pathway inhibitor in prostate cancer.

Studies evaluating the clinical relevance of individual HSPs in prostate cancer have been limited to small patient cohorts and lack comprehensive interrogation of lethal disease (27). To address this, and the fact that multiple HSPs and HSF1 have been implicated in AR signaling, we explored the clinical relevance of the GO cellular response to heat gene expression signature (which includes multiple HSPs and HSF1) in advanced prostate cancer (39, 61). These studies demonstrate across multiple transcriptome cohorts of patients with mCRPC that the GO cellular response to heat gene expression signature associates with previously derived AR and AR-V7 signatures as well as worse clinical outcomes (3, 14, 51, 52). These data further support targeting HSPs to overcome persistent AR signaling and prostate cancer treatment resistance.

In light of these challenges, and to support the development of novel therapeutic strategies to exploit this important prostate cancer biological feature, we evaluated NXP800, a clinical-stage drug optimized from a hit in a phenotypic screen aimed to discover HSF1 pathway inhibitors (based on the ability to block HSP90 inhibitor-mediated HSP72 induction), which is currently being evaluated in a phase Ib clinical trial in ovarian cancer (NCT05226507; refs. 40, 43). The studies herein demonstrate that NXP800 reduced basal and HSF1-mediated, HSP90 inhibitor–induced, HSP72 protein expression levels in prostate cancer cell lines, consistent with its mechanistic development rationale and preclinical therapeutic activity in sensitive preclinical models of ovarian cancer (40, 70). Importantly, NXP800 inhibited AR/AR-V7 transactivation and reduced AR DNA binding and expression of AR-responsive genes, as well as decreased the growth of prostate cancer cell lines and patient-derived models with common ARSI-resistant mechanisms, including AR amplification and AR-V7 protein expression. These data support the consideration of NXP800 for prostate cancer–specific clinical development.

Interestingly, NXP800 demonstrated anticancer activity in AR-negative prostate cancer cell lines, suggesting that the antitumor activity of the drug is not only due to blocking AR signaling. Unbiased analyses of RNA-seq data across multiple prostate cancer cell lines treated with NXP800 demonstrated a predominant increase in the UPR gene signature. Further orthogonal validation of this identified that NXP800 increased eIF2α phosphorylation and ATF4, ATF6, and IRE1 protein expression. It is important to note that the eIF2α axis has been previously shown to regulate ATF4, ATF6, and IRE1 protein expression, and although we have not fully interrogated the activation status of the three arms of the UPR, we have identified that the NXP800-mediated phenotype may be driven through the eIF2α axis, and this warrants further investigation in the future (71, 72). Considering those pathways beyond the UPR, RNA-seq analyses demonstrated that treatment with NXP800 impacted other key pathways, including AR, MYC, and E2F activity, implicated in prostate cancer treatment resistance (63, 66, 73). These data also support the study of NXP800 in subjects suffering from mCRPC.

Importantly, activation of the UPR has previously been reported to be both beneficial and detrimental to the survival of cancer cells (74–78). Although the UPR is generally viewed as protective for prostate cancer cells, treatment with ONC201, a UPR activator, led to the induction of the UPR and cell death in prostate cancer models, which is consistent with our studies (74, 77, 78). Considering these data, we next investigated whether the NXP800-mediated increase in eIF2α phosphorylation and ATF4, ATF6, and IRE1 protein expression was beneficial or detrimental to these prostate cancer models studied. To do this, we used the small-molecule eIF2B stabilizer/activator ISRIB to determine whether the NXP800-induced phenotype can be rescued (67). ISRIB did rescue some of the impact of NXP800-mediated increases in components of the UPR, including ATF4, ATF6, and IRE1 protein expression, which caused a reduction of growth inhibition across most of our models. In addition, NXP800-induced suppression of AR-responsive genes, *E2F1* to *E2F3* RNA expression, and E2F1 protein expression was attenuated by ISRIB, consistent with our hypothesis that these changes, including ATF4, ATF6, and IRE1 protein induction, may be driven by NXP800-mediated activation of the eIF2α axis (71, 72). It will be important that follow-on studies deconvolute the mechanism by which NXP800 activates the eIF2α axis, including investigation of the eIF2α kinases (such as PERK, PKR, GCN2, and HRI), and interrogate whether NXP800 activates the IRE1 and ATF6 pathways, which may all play a role in the observed NXP800-mediated phenotype, as this will further support the clinical development of this therapeutic strategy (79).

Consistent with our ISRIB studies, NXP800 had limited to no impact on eIF2α phosphorylation and ATF4, ATF6 and IRE1 protein expression, or effects on AR, E2F and MYC in our NXP800-resistant 22Rv1 prostate cancer subline, further supporting that activation of the eIF2α axis plays a role in the NXP800-mediated phenotype observed. Overall, these data suggest that NXP800, through a novel mechanism of action, offers the potential for clinical antitumor activity against prostate tumors not only with AR splice variant expression but also with high E2F and MYC activity, supporting clinical evaluation following progression on the first AR-targeting therapy in which both AR-dependent and AR-independent mechanisms of resistance have emerged (6–14, 80).

The work herein does have limitations that we acknowledge. The translation of therapeutic strategies targeting HSPs and proteostasis to the clinic has been challenged by pharmacologic and tolerability limitations, which may be relevant to NXP800, although the oral HSP90 inhibitor pimitespib has been approved in Japan (35). Predictive biomarkers of response to our proposed new therapeutic strategy also now have to be developed, and we have not pursued this to date, even though we envision that it may include patients with tumors exhibiting AR-V7 expression, high activating E2Fs, and/or high MYC. In addition, our *in vivo* studies, although supported by multiple *in vitro* prostate cancer cell line and patient-derived prostate cancer models, remain limited. Our clinical RNA-seq data focused on signatures derived from CRPC transcriptome cohorts, and we did not explore expression of single HSPs at the RNA or protein level. However, this removed the challenges of preanalytic variables that can confound IHC analyses and supported the identification of signatures that provide a more accurate representation of cellular processes that are rarely represented by single autonomous molecules. The development and interrogation of our NXP800-resistant 22Rv1 prostate cancer subline has not identified the mechanism of acquired resistance to NXP800 despite the near complete reversal of the NXP800-mediated phenotype. However, our investigative studies were limited to RNA-seq analyses, and these models provide a valuable resource for further mechanistic work to investigate drivers of NXP800 resistance and identify rational synergistic combinations to further support the clinical development of NXP800 in lethal prostate cancer. Critically, activation of the UPR can be beneficial and detrimental to prostate cancer cells, and although we and others have confirmed that this can drive prostate cancer cell death, as this may be context specific, it will be important that further studies elucidate those specific tumors that will respond to such therapeutic strategies (74, 77, 78). Importantly, we have identified pharmacodynamic markers, such as AR, AR-V7, PSA, phospho-eIF2α, E2F1, and HSP72, as well as gene expression signatures that can be incorporated into clinical pharmacokinetic/pharmacodynamic studies as part of a Pharmacological Audit Trail (81).

Taken together, these data, alongside other studies that have interrogated HSF1 pathway inhibition in prostate cancer models, support the biological and clinical relevance of HSPs in mCRPC. Importantly, this study with NXP800, a clinical-stage developmental HSF1 pathway inhibitor currently being trialed in ovarian cancer, supports its clinical evaluation as a novel therapeutic strategy for patients suffering from mCRPC, whose tumors have evidence of spliced AR, increased E2F1 to E2F3, and MYC function (42).

## Supplementary Material

Supplementary Figure S1 Supplementary figure 1: Chemical structures of NXP800 and CCT365248

Supplementary Figure S2 Supplementary figure 2: AR and AR-V7 bind members of the 70KDa heat shock protein family and heat shock mediated cellular stress increases HSP72 and AR-V7 protein expression, and associates with GO Cellular Response to Heat gene expression signature, in PCa cells

Supplementary Figure S3 Supplementary figure 3: GO Cellular Response to Heat gene expression signature associates with AR signaling in CRPC transcriptomes

Supplementary Figure S4 Supplementary figure 4: NXP800 decreases basal HSP72 protein levels and blocks HSP72 protein induction in response to HSP90 inhibition in PCa cell lines

Supplementary Figure S5 Supplementary figure 5: Enzalutamide inhibits AR signaling in enzalutamide responsive VCaP PCa cells but not enzalutamide resistant LNCaP95 and 22Rv1 PCa cells.

Supplementary Figure S6 Supplementary figure 6: Inhibition of the unfolded protein response with ISRIB rescues NXP800-mediated suppression of AR signaling and PCa model growth

Supplementary Figure S7 Supplementary figure 7: NXP800 does not decrease basal HSP72 protein levels and HSP90 inhibitor-induced HSP72 protein induction in NXP800-resistant 22Rv1 PCa cell sub-lines

Supplementary Figure S8 Supplementary figure 8: NXP800 does not further impact AR transactivation or AR signaling in NXP800-resistant 22Rv1 PCa cell sub-lines.

Supplementary Figure S9 Supplementary figure 9: NXP800 demonstrates tolerability in a castration-resistant VCaP PCa cell line-derived mouse xenograft

Supplementary Figure S10 Supplementary figure 10: NXP800 demonstrates limited impact on AR and AR-V7 protein levels and associated AR signaling in-vivo but does induce apoptosis and reduce cellular proliferation

Supplementary Tables S1-S13 Supplementary Tables
