# Supplementary figures and images for: NXP800 Activates the Unfolded Protein Response, Altering AR and E2F Function to Impact Castration-Resistant Prostate Cancer Growth
Source: Clin Cancer Res. 2025 Jan 9;31(6):1109–26. doi: 10.1158/1078-0432.CCR-24-2386 (PMC11911806; doi:10.1158/1078-0432.CCR-24-2386)

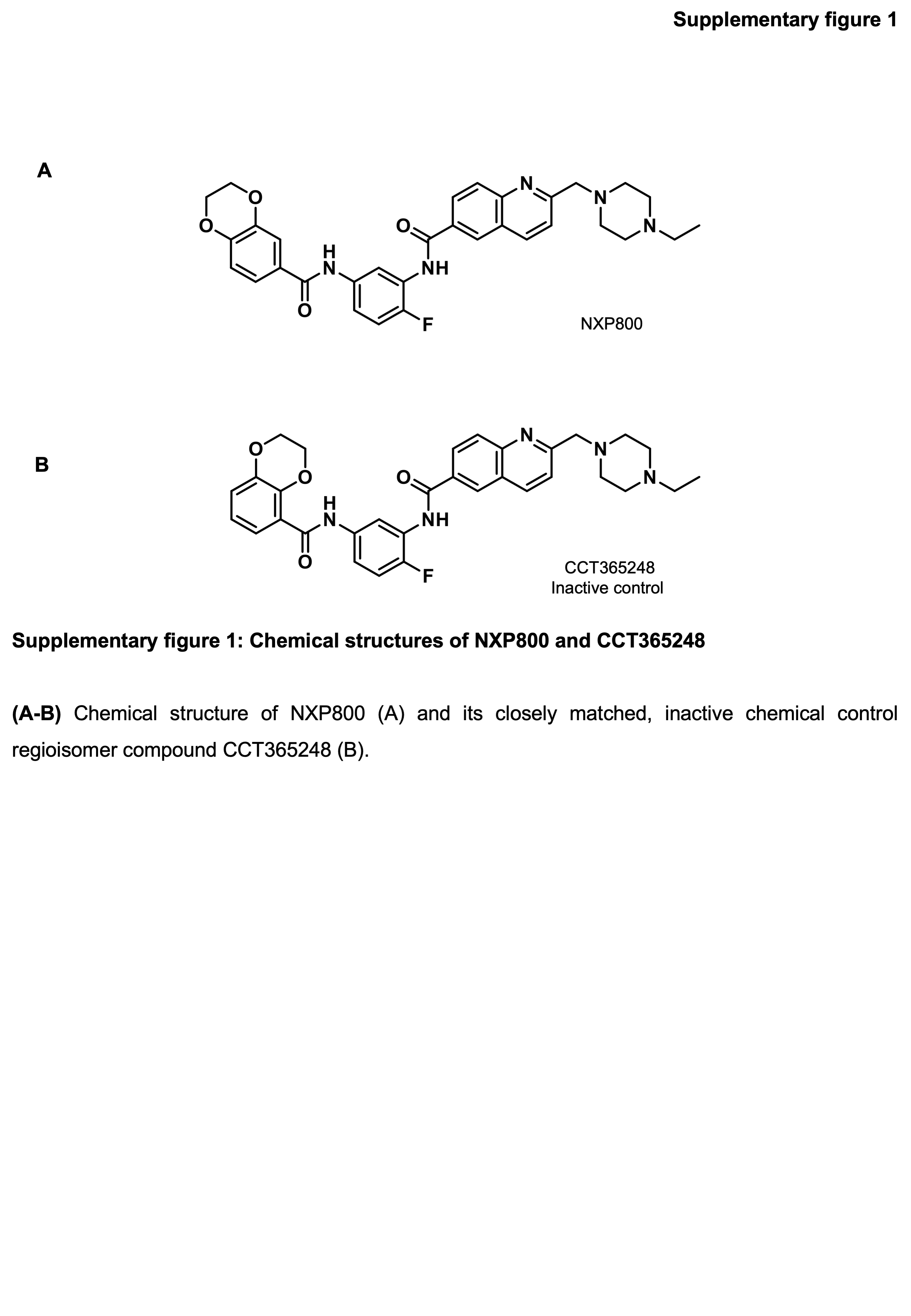

Supplement: Supplementary Figure S1 — Supplementary figure 1: Chemical structures of NXP800 and CCT365248 [file ccr-24-2386_supplementary_figure_s1_suppfs1.png]

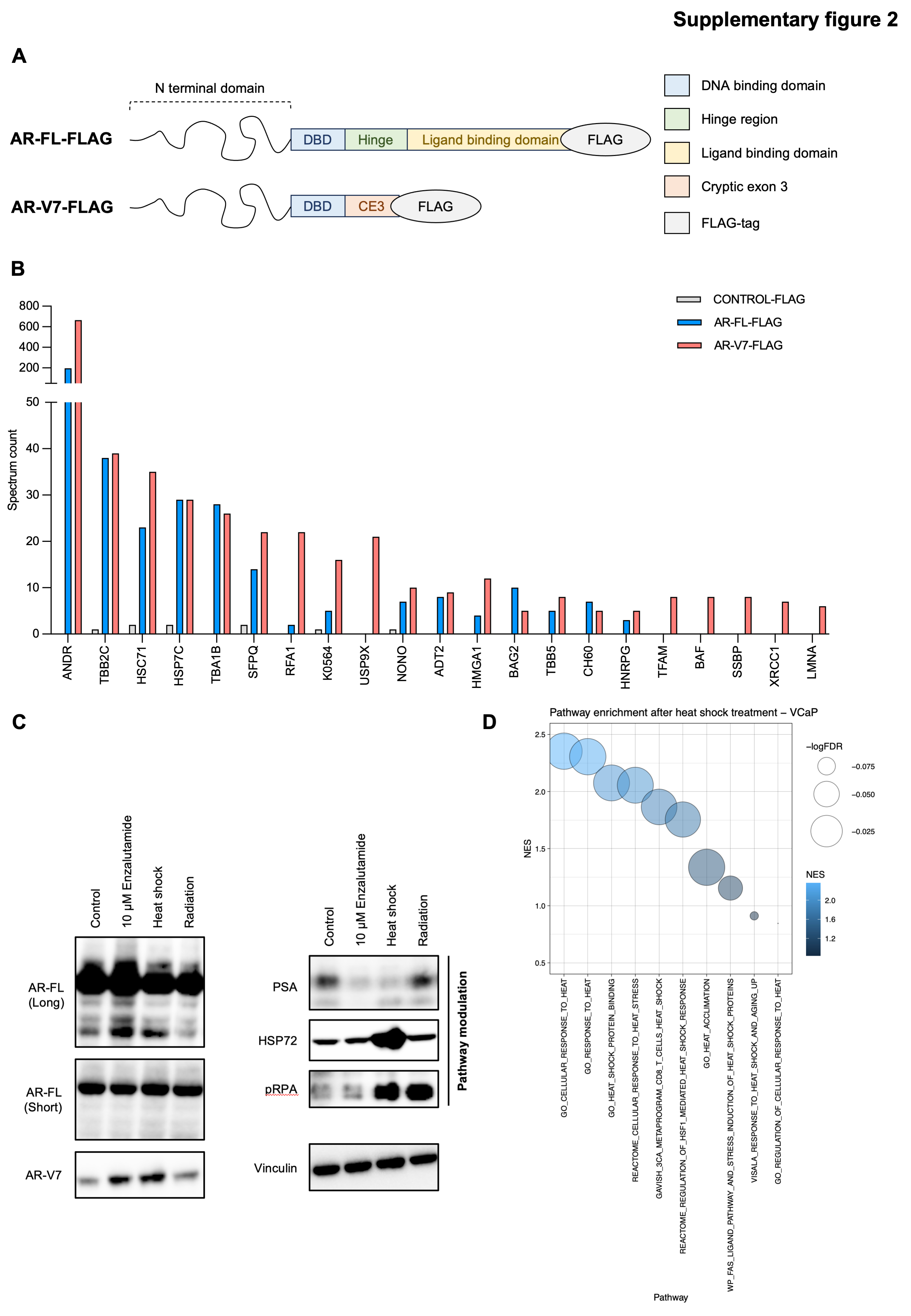

Supplement: Supplementary Figure S2 — Supplementary figure 2: AR and AR-V7 bind members of the 70KDa heat shock protein family and heat shock mediated cellular stress increases HSP72 and AR-V7 protein expression, and associates with GO Cellular Response to Heat gene expression signature, in PCa cells [file ccr-24-2386_supplementary_figure_s2_suppfs2.png]

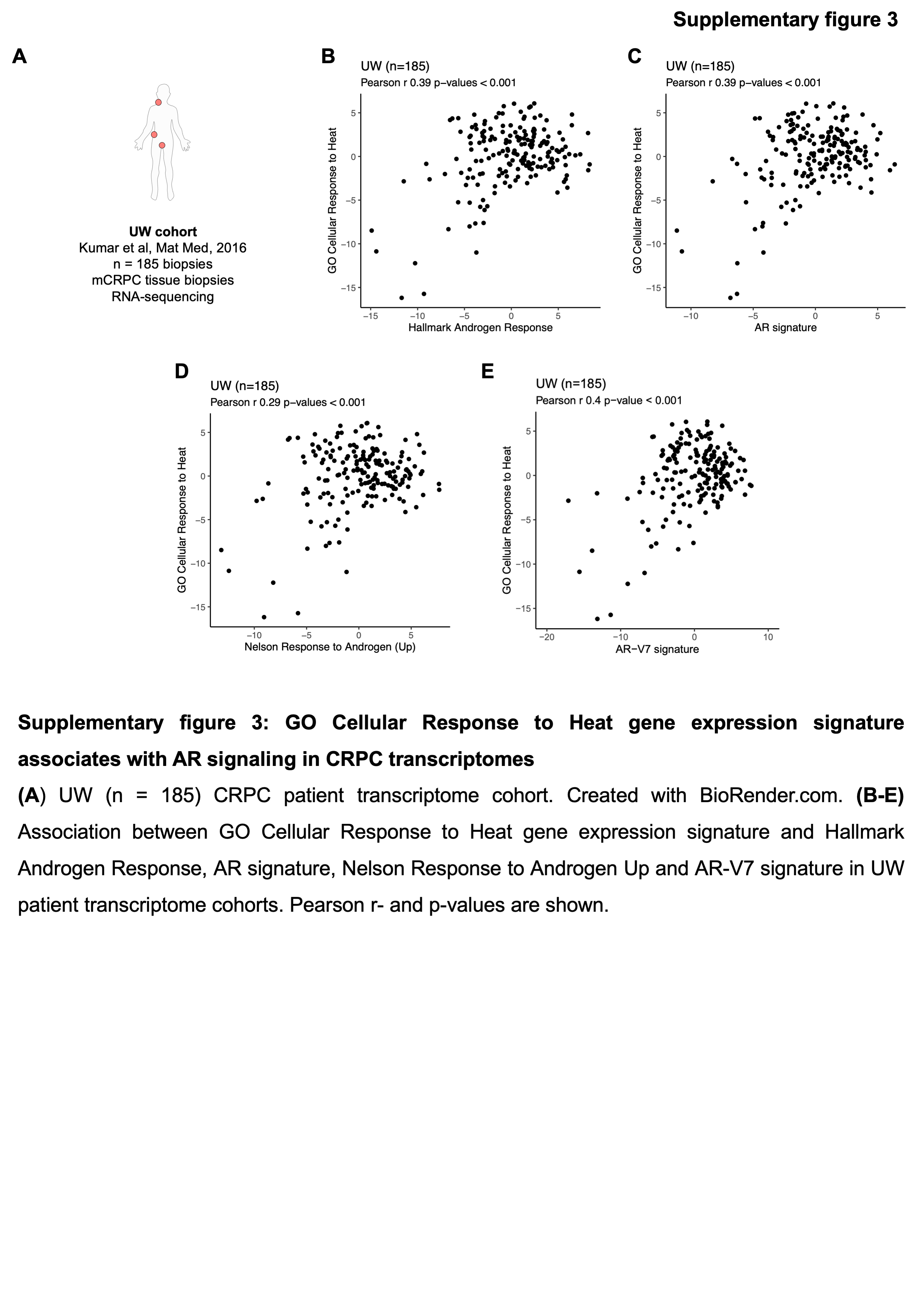

Supplement: Supplementary Figure S3 — Supplementary figure 3: GO Cellular Response to Heat gene expression signature associates with AR signaling in CRPC transcriptomes [file ccr-24-2386_supplementary_figure_s3_suppfs3.png]

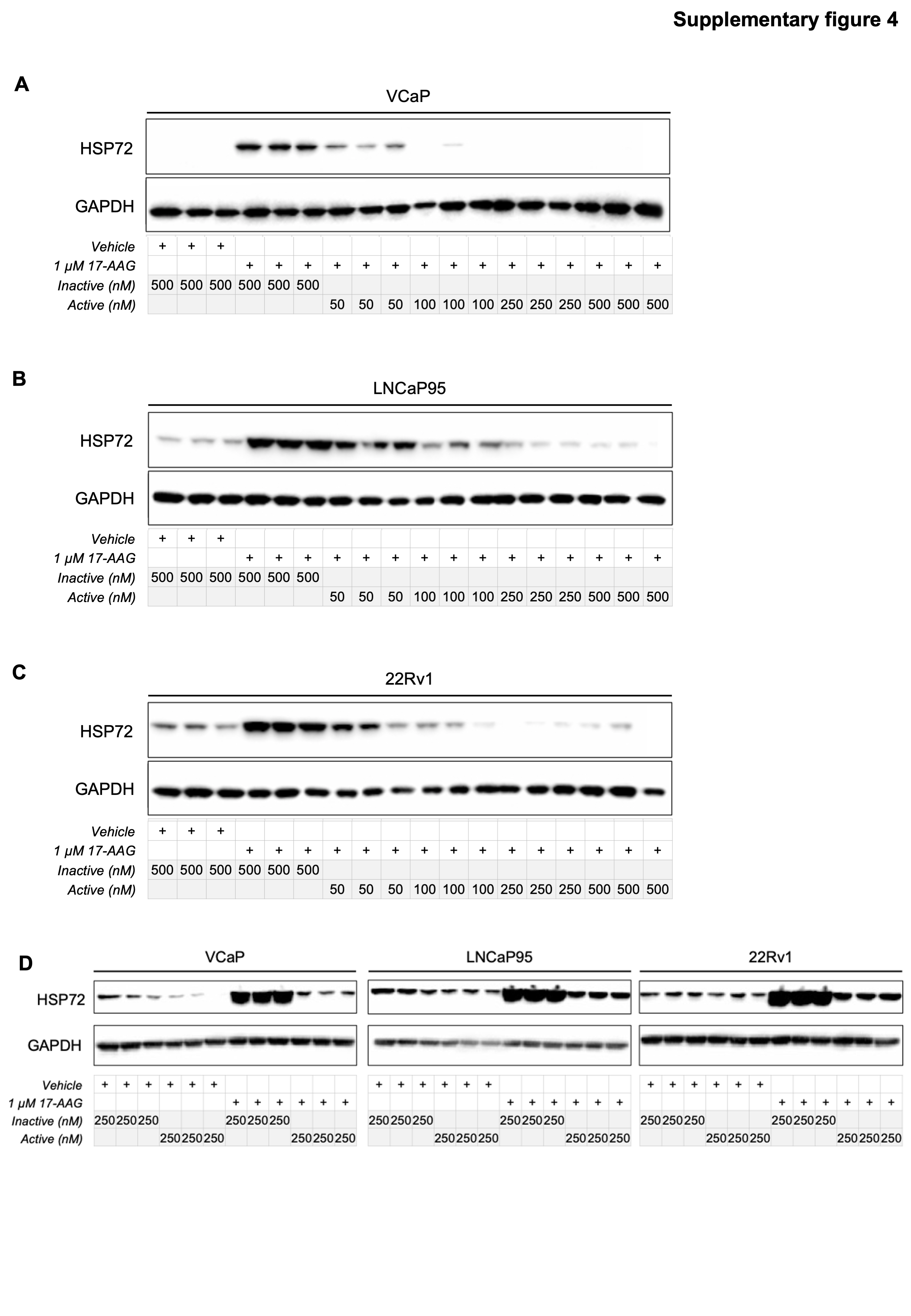

Supplement: Supplementary Figure S4 — Supplementary figure 4: NXP800 decreases basal HSP72 protein levels and blocks HSP72 protein induction in response to HSP90 inhibition in PCa cell lines [file ccr-24-2386_supplementary_figure_s4_suppfs4.png]

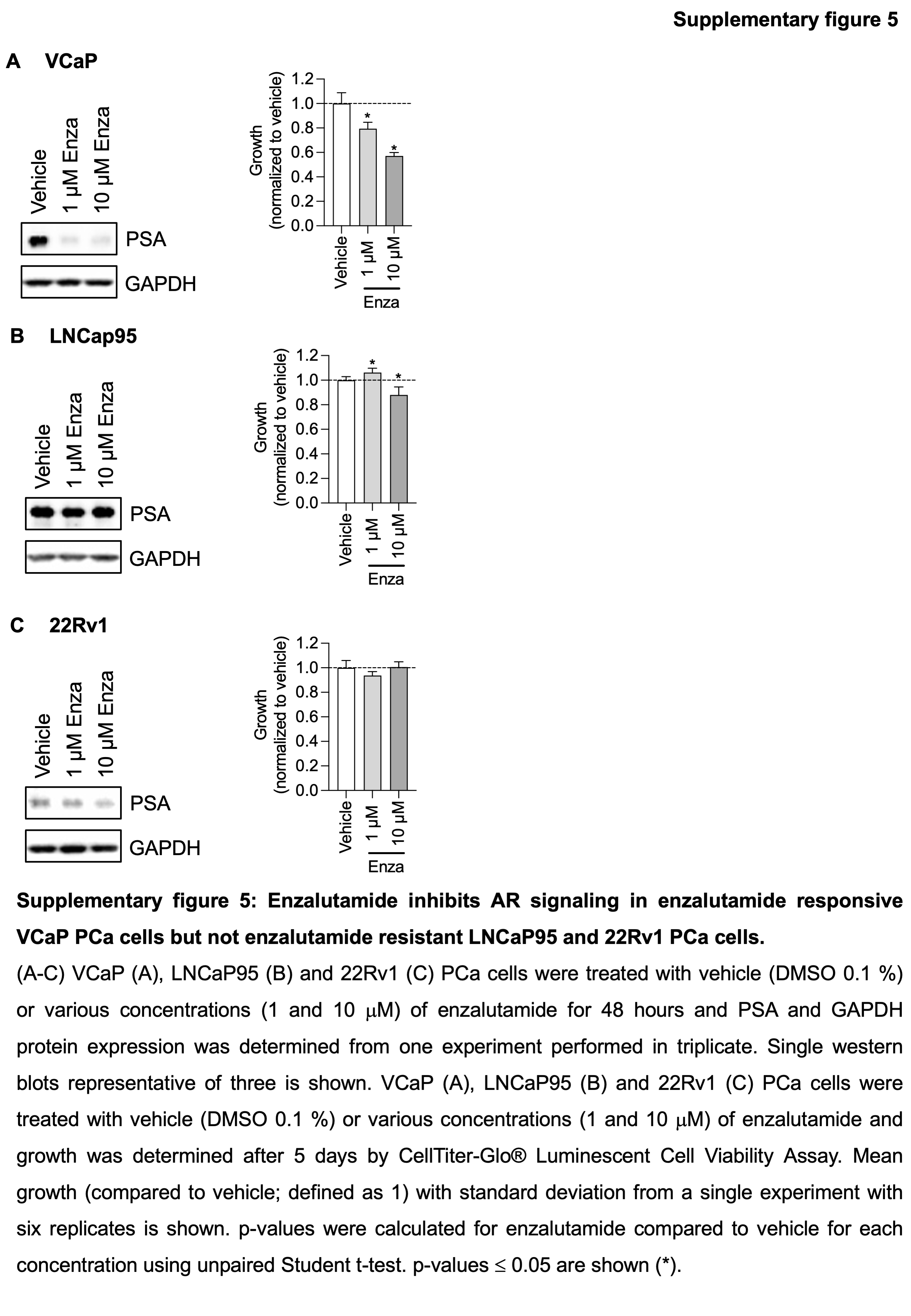

Supplement: Supplementary Figure S5 — Supplementary figure 5: Enzalutamide inhibits AR signaling in enzalutamide responsive VCaP PCa cells but not enzalutamide resistant LNCaP95 and 22Rv1 PCa cells. [file ccr-24-2386_supplementary_figure_s5_suppfs5.png]

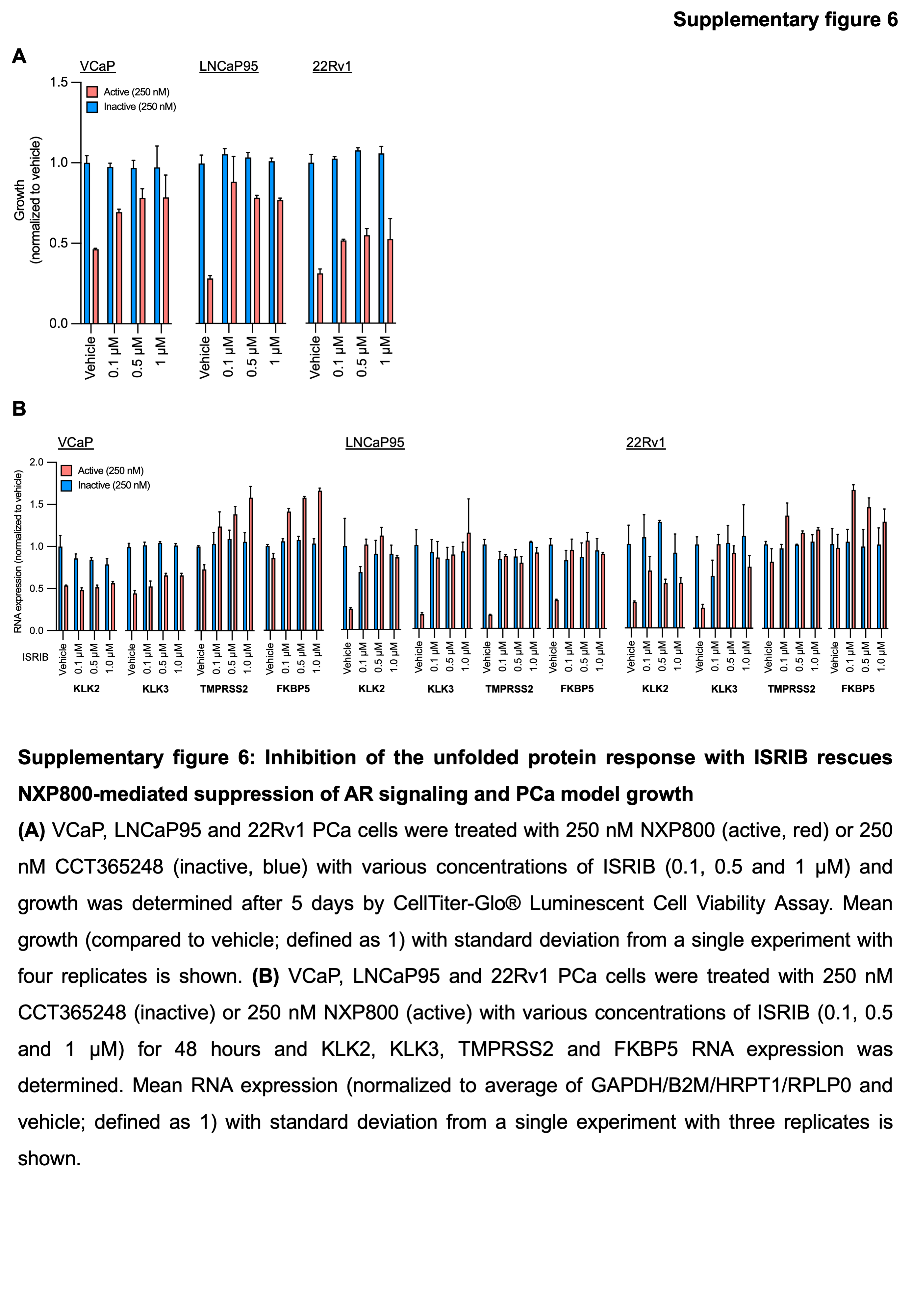

Supplement: Supplementary Figure S6 — Supplementary figure 6: Inhibition of the unfolded protein response with ISRIB rescues NXP800-mediated suppression of AR signaling and PCa model growth [file ccr-24-2386_supplementary_figure_s6_suppfs6.png]

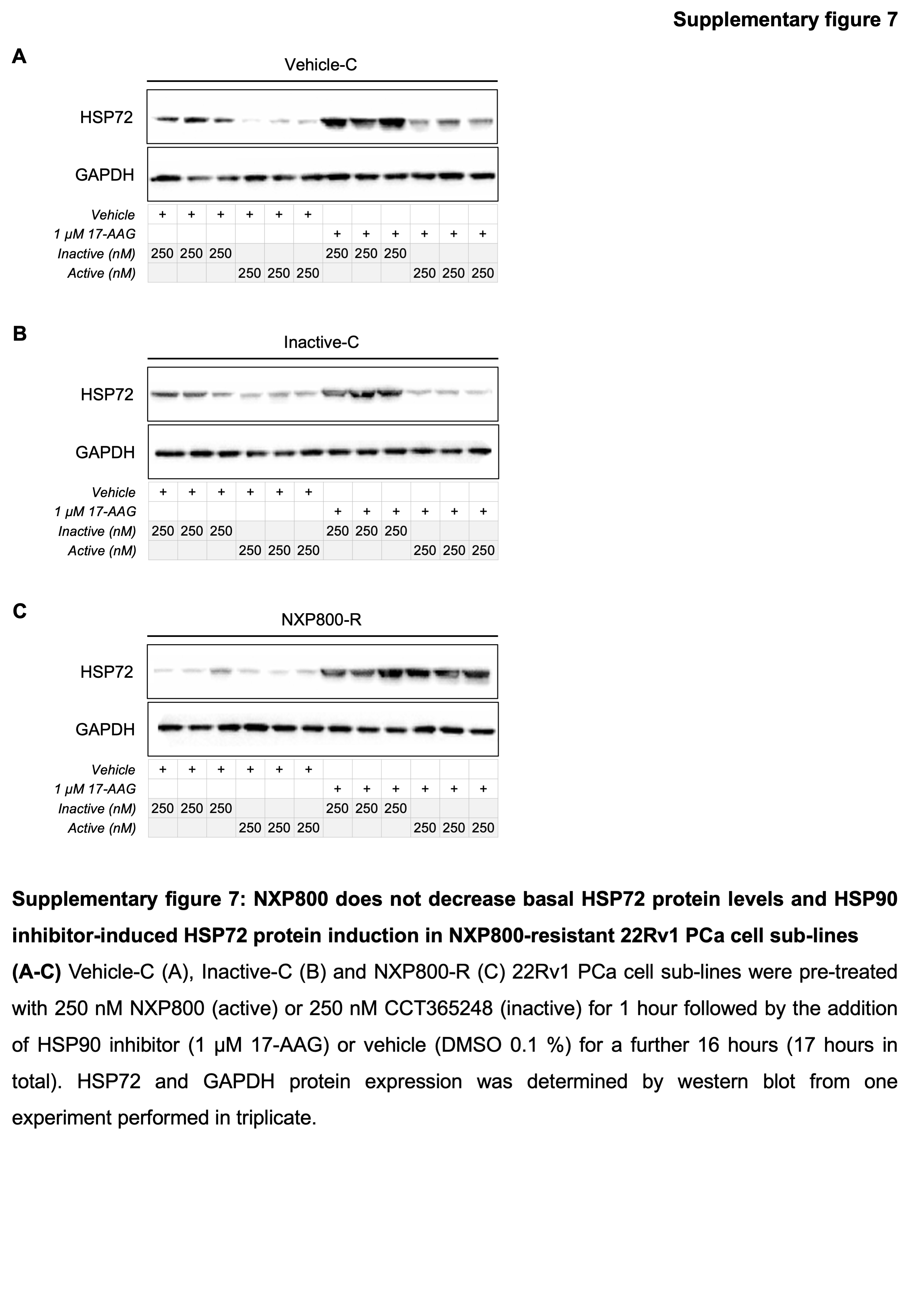

Supplement: Supplementary Figure S7 — Supplementary figure 7: NXP800 does not decrease basal HSP72 protein levels and HSP90 inhibitor-induced HSP72 protein induction in NXP800-resistant 22Rv1 PCa cell sub-lines [file ccr-24-2386_supplementary_figure_s7_suppfs7.png]

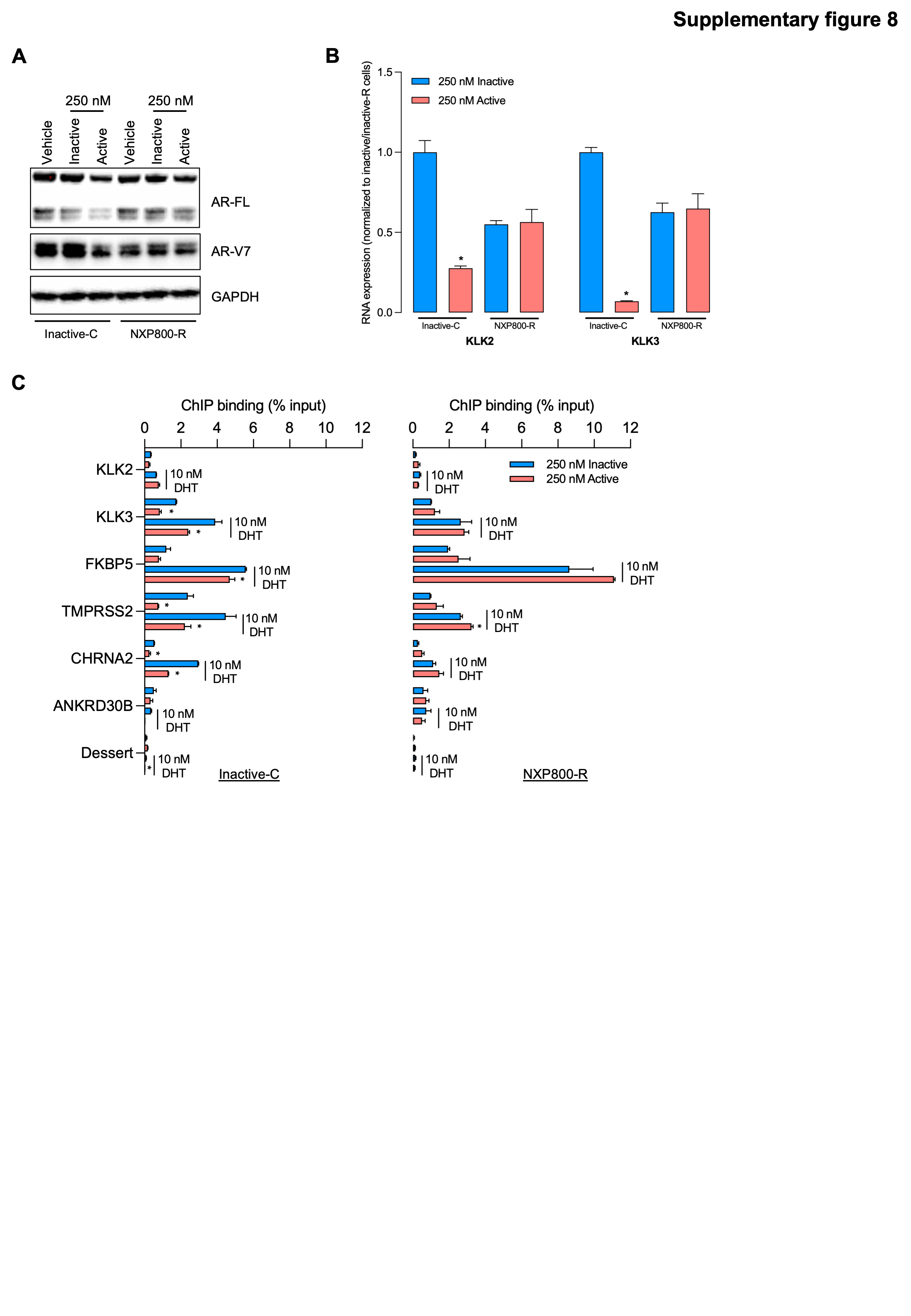

Supplement: Supplementary Figure S8 — Supplementary figure 8: NXP800 does not further impact AR transactivation or AR signaling in NXP800-resistant 22Rv1 PCa cell sub-lines. [file ccr-24-2386_supplementary_figure_s8_suppfs8.png]

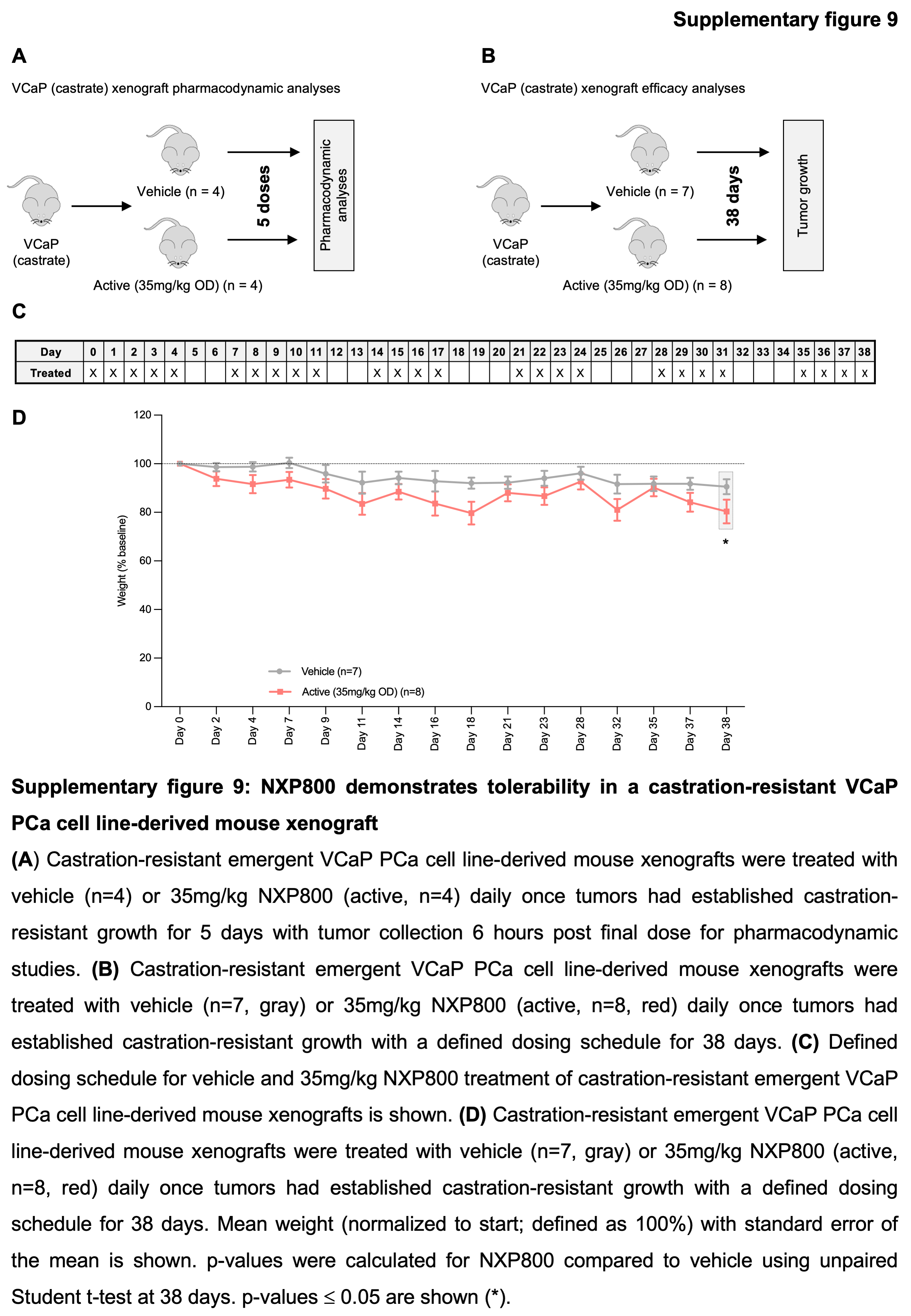

Supplement: Supplementary Figure S9 — Supplementary figure 9: NXP800 demonstrates tolerability in a castration-resistant VCaP PCa cell line-derived mouse xenograft [file ccr-24-2386_supplementary_figure_s9_suppfs9.png]

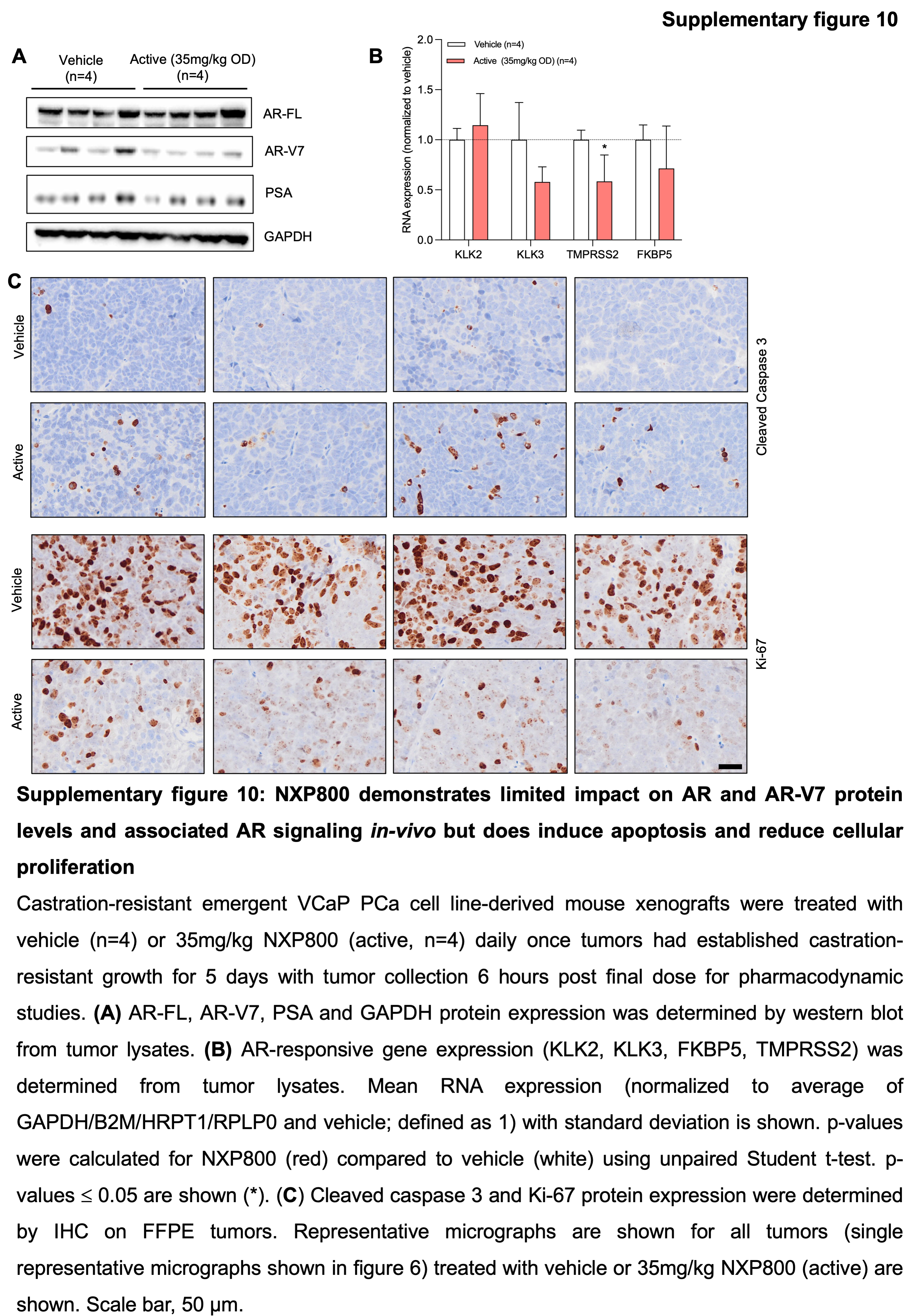

Supplement: Supplementary Figure S10 — Supplementary figure 10: NXP800 demonstrates limited impact on AR and AR-V7 protein levels and associated AR signaling in-vivo but does induce apoptosis and reduce cellular proliferation [file ccr-24-2386_supplementary_figure_s10_suppfs10.png]
