# Supplementary material for: NXP800 Activates the Unfolded Protein Response, Altering AR and E2F Function to Impact Castration-Resistant Prostate Cancer Growth
Source: Clin Cancer Res. 2025 Jan 9;31(6):1109–26. doi: 10.1158/1078-0432.CCR-24-2386 (PMC11911806; doi:10.1158/1078-0432.CCR-24-2386)
Supplement: Supplementary Tables S1-S13 — Supplementary Tables [file ccr-24-2386_supplementary_tables_s1-s13_suppts1-ts13.docx]

**Supplementary tables**

| **Cell line** | **Supplier** | **Catalogue ID** | **Media^** | **Serum (10%)** |
| --- | --- | --- | --- | --- |
| 22Rv1 | ATCC | CRL-2505, RRID:CVCL_1045 | RMPI | FBS |
| VCaP | ATCC | CRL-2876, RRID:CVCL_2235 | DMEM | FBS |
| LNCaP | ATCC | CRL-1740, RRID:CVCL_1379 | RMPI | FBS |
| LNCaP95 | Dr Meeker/Dr Luo* | RRID:CVCL_ZC87 | RMPI^ | CSS |
| DU145 | ATCC | HTB-81, RRID:CVCL_0105 | DMEM | FBS |
| PC3 | ATCC | CRL-1345, RRID:CVCL_0035 | F12 HAM | FBS |

**Supplementary table 1: Cell lines**

ATCC – American Type Culture Collection, RPMI – Roswell Park Memorial Institute 1640 Medium, DMEM – Dulbecco’s Modified Eagle Medium, FBS – fetal bovine serum, CSS – charcoal stripped serum, * - LNCaP95 cells were a kind gift from Drs. Alan K Meeker and Jun Luo (Johns Hopkins University, Baltimore, Maryland, USA), ^ - phenol red free.

| **Compound** | **Supplier** | **Catalogue ID** |
| --- | --- | --- |
| 17-AGG | Sigma-Aldrich | A476, |
| Enzalutamide | Selleckchem | S1250 |
| Puromycin | Thermo Fisher Scientific | A1113803 |
| ISRIB | Sigma-Aldrich | SML0843 |
| CCT365248 | Kind gifts from the Centre for Cancer Drug Discovery, Division of Cancer Therapeutics at The Institute of Cancer Research | |
| NXP800 |  |  |

**Supplementary table 2: Compounds**

| POM121C | NUP160 | HSPD1 | MAPK1 | YWHAE |
| --- | --- | --- | --- | --- |
| PDCD6 | SIRT1 | DNAJB1 | MAPK3 | NUP37 |
| DNAJB6 | NUP188 | NUP43 | TCIM | PDCL3 |
| CDKN1A | NUP62 | IL1A | NUP107 | NUP85 |
| STUB1 | MTOR | CXCL10 | BAG1 | NUP214 |
| SLU7 | HSPB8 | IRAK1 | PTGS2 | AAAS |
| CETN1 | MYOF | HSP90AB2P | RPTOR | CAMK2A |
| PTGES3 | CHORDC1 | LYN | CCAR2 | CLPB |
| NUP50 | DNAJC2 | MAPT | RANBP2 | CAMK2B |
| ARPP21 | HTRA2 | ATXN3 | RBBP7 | CAMK2D |
| HSPH1 | SCARA5 | HSBP1L1 | TRPV4 | CAMK2G |
| NUP42 | GSK3B | ATM | RPA1 | SEH1L |
| SLC52A3 | HDAC2 | NF1 | RPA2 | AKT1S1 |
| NUP35 | HMOX1 | ATP2A2 | RPA3 | RAE1 |
| CREBBP | HSBP1 | NUP88 | SEC13 | MAPKAPK2 |
| CRYAB | HSF1 | NUP98 | MLST8 | CD34 |
| DAXX | HSPA1A | IER5 | ST8SIA1 | BAG5 |
| DHX36 | HSPA1B | HIKESHI | HSP90AB4P | BAG4 |
| EIF2S1 | HSPA1L | NUP54 | STAC | BAG3 |
| EP300 | HSPA6 | POLR2D | THBS1 | BAG2 |
| FGF1 | HSPA8 | ATR | TPR | NUP155 |
| TFEC | HSP90AA1 | ANO1 | DNAJC7 | NUP93 |
| FKBP4 | HSP90AA4P | PRKACA | SUMO1 | NUP58 |
| NUP205 | HSP90AB1 | NDC1 | VCP | POM121 |
| NUP210 | HSP90AB3P | NUP133 | TRPV1 | NUP153 |

**Supplementary table 3: Genes included in the GO Cellular Response to Heat gene expression signature (GO:0034605)**

| **Protein target** | **Supplier** | **Catalogue ID** |
| --- | --- | --- |
| AR-FL | Agilent Dako | M3562, RRID:AB_2060174 |
| AR-V7 | RevMab | 31-1109-00, RRID:AB_2716436 |
| PSA | Cell Signaling | 2475, RRID:AB_2797601 |
| GAPDH | Santa Cruz biotechnology | sc-32233, RRID:AB_627679 |
| HSP70/HSP72 | Enzo life sciences | ADI-SPA-810, RRID:AB_10616513 |
| PERK | Cell Signaling | 3192, RRID:AB_2095847 |
| Phospho-eIF2⍺ | Cell Signaling | 3398, RRID:AB_2096481 |
| Total eIF2⍺ | Cell Signaling | 5324, RRID:AB_10692650 |
| ATF4 | Cell Signaling | 11815, RRID:AB_2616025 |
| ATF6 | Cell Signaling | 65880, RRID:AB_2799696 |
| IRE1 | Cell Signaling | 3294, RRID:AB_823545 |
| Puromycin | MERCK | MABE343, RRID:AB_3099686 |
| Phospho-RPA | abcam | ab109394, RRID:AB_10860648 |
| Vinculin | Sigma-Aldrich | V9131, RRID:AB_477629 |
| E2F1 | Cell Signaling | 3742, RRID:AB_2096936 |

**Supplementary table 4:** **Antibodies used for western blot analyses**

| **Gene target** | **Supplier** | **Catalogue ID** |
| --- | --- | --- |
| KLK2 | Thermo Fisher Scientific | Hs00428384_g1 |
| KLK3 |  | Hs02576345_m1 |
| TMPRSS2 |  | Hs01122322_m1 |
| FKBP5 |  | Hs01561006_m1 |
| GAPDH |  | Hs02786624_g1 |
| B2M |  | Hs00187842_m1 |
| HRPT1 |  | Hs02800695_m1 |
| RPLP0 |  | Hs00420895_gH |

**Supplementary table 5:** **TaqMan probes for qRT-PCR analyses**

| **Gene target** | **Forward** | **Reverse** |
| --- | --- | --- |
| KLK2 | ACCCCTGTTGCTGTTCATCCTG | CCGCCCTTGCCCTGTTGG |
| KLK3 | ACAGACCTACTCTGGAGGAAC | AGACAAGGGTGGAAGGCTCT |
| TMPRSS2 | CCAGGGAAGGAAGGAACACC | CTGACTTGGGCACACGGAAT |
| FKBP5 | GCGAGCTGCAAAACATCACT | GTGCCAGCCACATTCAGAAC |
| ANKRD30B | TCAAAGTCAACCAAGAGGACTCA | ACGGCAGGCTAAATGGGTTT |
| CHRNA2 | GTCCAGTCACCACGATGCT | GCAGCTCTGTCCTTACTCCAA |
| Desert (negative control) | CTAGGAGGGTGGAGGTAGGG | GCCCCAAACAGGAGTAATGA |

**Supplementary table 6:** **Primers for ChIP-PCR analysis**

|  |  | **VCaP** | | | |
| --- | --- | --- | --- | --- | --- |
|  |  | **100 nM** | | **250 nM** | |
| **HALLMARK** |  | **NES** | **FDR** | **NES** | **FDR** |
| HALLMARK_UNFOLDED_PROTEIN_RESPONSE |  | 1.61 | 0.0232 | 1.86 | 0.0005 |
| HALLMARK_TNFA_SIGNALING_VIA_NFKB |  | -0.76 | 0.7547 | 1.06 | 0.4165 |
| HALLMARK_HEDGEHOG_SIGNALING |  | 0.61 | 0.7547 | 0.78 | 0.6676 |
| HALLMARK_KRAS_SIGNALING_UP |  | 1.16 | 0.4183 | 1.13 | 0.2719 |
| HALLMARK_TGF_BETA_SIGNALING |  | 0.78 | 0.7547 | 1.05 | 0.4620 |
| HALLMARK_P53_PATHWAY |  | 0.69 | 0.7547 | 0.74 | 0.7120 |
| HALLMARK_HEME_METABOLISM |  | 0.51 | 0.7547 | 0.81 | 0.6817 |
| HALLMARK_HYPOXIA |  | 1.08 | 0.4921 | 1.20 | 0.1908 |
| HALLMARK_COAGULATION |  | 0.87 | 0.6967 | 0.95 | 0.5345 |
| HALLMARK_ALLOGRAFT_REJECTION |  | 1.04 | 0.5439 | 0.99 | 0.5193 |
| HALLMARK_PROTEIN_SECRETION |  | -0.57 | 0.7547 | 0.85 | 0.6465 |
| HALLMARK_INFLAMMATORY_RESPONSE |  | 0.93 | 0.6253 | 1.39 | 0.0668 |
| HALLMARK_IL6_JAK_STAT3_SIGNALING |  | 0.77 | 0.7547 | 0.95 | 0.5345 |
| HALLMARK_MYOGENESIS |  | 1.08 | 0.4921 | 1.03 | 0.4620 |
| HALLMARK_EPITHELIAL_MESENCHYMAL_TRANSITION |  | -1.03 | 0.5439 | 1.14 | 0.2624 |
| HALLMARK_XENOBIOTIC_METABOLISM |  | 1.22 | 0.3267 | 1.16 | 0.2543 |
| HALLMARK_PI3K_AKT_MTOR_SIGNALING |  | -1.02 | 0.5439 | -0.87 | 0.6262 |
| HALLMARK_UV_RESPONSE_DN |  | 0.55 | 0.7547 | 0.77 | 0.7038 |
| HALLMARK_WNT_BETA_CATENIN_SIGNALING |  | 0.68 | 0.7547 | 0.52 | 0.7120 |
| HALLMARK_APICAL_JUNCTION |  | 0.71 | 0.7547 | 0.84 | 0.6817 |
| HALLMARK_APOPTOSIS |  | -1.06 | 0.4921 | -1.00 | 0.5148 |
| HALLMARK_COMPLEMENT |  | -1.05 | 0.4921 | -0.95 | 0.5345 |
| HALLMARK_APICAL_SURFACE |  | -0.99 | 0.597 | -1.45 | 0.1611 |
| HALLMARK_IL2_STAT5_SIGNALING |  | 0.6 | 0.7547 | -0.80 | 0.6817 |
| HALLMARK_PANCREAS_BETA_CELLS |  | 0.97 | 0.6186 | -1.30 | 0.2640 |
| HALLMARK_INTERFERON_GAMMA_RESPONSE |  | -1.01 | 0.5439 | -1.24 | 0.1857 |
| HALLMARK_KRAS_SIGNALING_DN |  | 1.33 | 0.1621 | 1.26 | 0.1746 |
| HALLMARK_INTERFERON_ALPHA_RESPONSE |  | -1.41 | 0.1019 | -1.51 | 0.0691 |
| HALLMARK_SPERMATOGENESIS |  | -2.01 | 0.0007 | -2.01 | 0.0004 |
| HALLMARK_DNA_REPAIR |  | -2.04 | 2E-05 | -1.90 | 0.0001 |
| HALLMARK_NOTCH_SIGNALING |  | -0.92 | 0.6193 | -1.02 | 0.5066 |
| HALLMARK_UV_RESPONSE_UP |  | -0.86 | 0.7547 | -0.96 | 0.5345 |
| HALLMARK_REACTIVE_OXYGEN_SPECIES_PATHWAY |  | 0.57 | 0.7547 | -1.00 | 0.5148 |
| HALLMARK_GLYCOLYSIS |  | -1.04 | 0.5019 | -0.95 | 0.5345 |
| HALLMARK_ADIPOGENESIS |  | -0.96 | 0.6193 | -1.08 | 0.4165 |
| HALLMARK_ESTROGEN_RESPONSE_EARLY |  | 0.56 | 0.7547 | -0.66 | 0.7120 |
| HALLMARK_ANGIOGENESIS |  | -0.93 | 0.6193 | -0.80 | 0.6401 |
| HALLMARK_BILE_ACID_METABOLISM |  | -1.47 | 0.0954 | -1.53 | 0.0448 |
| HALLMARK_MTORC1_SIGNALING |  | -1.51 | 0.0092 | -1.48 | 0.0270 |
| HALLMARK_PEROXISOME |  | -1.38 | 0.1317 | -1.37 | 0.1117 |
| HALLMARK_FATTY_ACID_METABOLISM |  | -1.11 | 0.4205 | -1.17 | 0.2640 |
| HALLMARK_CHOLESTEROL_HOMEOSTASIS |  | -1.37 | 0.1184 | -1.45 | 0.0691 |
| HALLMARK_ANDROGEN_RESPONSE |  | -1.87 | 0.0006 | -1.60 | 0.0134 |
| HALLMARK_MITOTIC_SPINDLE |  | -2.69 | 1E-09 | -2.39 | 1E-09 |
| HALLMARK_ESTROGEN_RESPONSE_LATE |  | -1.78 | 0.0007 | -1.63 | 0.0059 |
| HALLMARK_OXIDATIVE_PHOSPHORYLATION |  | -2.04 | 3E-06 | -1.92 | 6E-05 |
| HALLMARK_MYC_TARGETS_V2 |  | -1.79 | 0.0141 | -1.75 | 0.0105 |
| HALLMARK_MYC_TARGETS_V1 |  | -2.13 | 6E-08 | -2.06 | 1E-07 |
| HALLMARK_G2M_CHECKPOINT |  | -3.32 | 1E-09 | -3.14 | 1E-09 |
| HALLMARK_E2F_TARGETS |  | -3.38 | 1E-09 | -3.10 | 1E-09 |

**Supplementary table 7: Cellular pathways de-enriched and enriched in response to NXP800 treatment in VCaP PCa cells**

NES – normalized enrichment score, FDR – false discovery rate.

|  |  | **LNCaP95** | | | |
| --- | --- | --- | --- | --- | --- |
|  |  | **100 nM** | | **250 nM** | |
| **HALLMARK** |  | **NES** | **FDR** | **NES** | **FDR** |
| HALLMARK_UNFOLDED_PROTEIN_RESPONSE |  | 1.38 | 0.0941 | 1.18 | 0.2443 |
| HALLMARK_TNFA_SIGNALING_VIA_NFKB |  | 1.19 | 0.2398 | 1.24 | 0.2032 |
| HALLMARK_HEDGEHOG_SIGNALING |  | 1.42 | 0.1284 | 1.30 | 0.2032 |
| HALLMARK_KRAS_SIGNALING_UP |  | 1.38 | 0.0941 | 1.37 | 0.0901 |
| HALLMARK_TGF_BETA_SIGNALING |  | 1.03 | 0.4095 | 0.81 | 0.5928 |
| HALLMARK_P53_PATHWAY |  | 1.16 | 0.245 | 1.11 | 0.2875 |
| HALLMARK_HEME_METABOLISM |  | 1.17 | 0.2398 | 1.08 | 0.3391 |
| HALLMARK_HYPOXIA |  | 1.22 | 0.1815 | 1.22 | 0.2032 |
| HALLMARK_COAGULATION |  | 1.54 | 0.0438 | 1.56 | 0.0279 |
| HALLMARK_ALLOGRAFT_REJECTION |  | 1.38 | 0.1005 | 1.29 | 0.1805 |
| HALLMARK_PROTEIN_SECRETION |  | 1.07 | 0.3743 | 1.05 | 0.3712 |
| HALLMARK_INFLAMMATORY_RESPONSE |  | 1.71 | 0.0089 | 1.65 | 0.0088 |
| HALLMARK_IL6_JAK_STAT3_SIGNALING |  | 1.49 | 0.0675 | 1.56 | 0.0452 |
| HALLMARK_MYOGENESIS |  | 0.97 | 0.4463 | 1.01 | 0.4318 |
| HALLMARK_EPITHELIAL_MESENCHYMAL_TRANSITION |  | 1.49 | 0.0407 | 1.62 | 0.0129 |
| HALLMARK_XENOBIOTIC_METABOLISM |  | 1.14 | 0.2815 | 0.94 | 0.5149 |
| HALLMARK_PI3K_AKT_MTOR_SIGNALING |  | 0.59 | 0.6753 | 0.69 | 0.6733 |
| HALLMARK_UV_RESPONSE_DN |  | 0.84 | 0.5949 | 0.88 | 0.5489 |
| HALLMARK_WNT_BETA_CATENIN_SIGNALING |  | -1.02 | 0.4095 | -1.01 | 0.4312 |
| HALLMARK_APICAL_JUNCTION |  | 0.50 | 0.6753 | 0.60 | 0.6753 |
| HALLMARK_APOPTOSIS |  | 1.34 | 0.1090 | 1.20 | 0.2221 |
| HALLMARK_COMPLEMENT |  | 1.64 | 0.0089 | 1.50 | 0.0368 |
| HALLMARK_APICAL_SURFACE |  | 1.15 | 0.2995 | 1.30 | 0.2032 |
| HALLMARK_IL2_STAT5_SIGNALING |  | 0.95 | 0.4883 | 0.90 | 0.5453 |
| HALLMARK_PANCREAS_BETA_CELLS |  | 0.99 | 0.4368 | 0.97 | 0.4554 |
| HALLMARK_INTERFERON_GAMMA_RESPONSE |  | 0.90 | 0.5300 | 0.80 | 0.6271 |
| HALLMARK_KRAS_SIGNALING_DN |  | -1.17 | 0.2398 | -1.31 | 0.0743 |
| HALLMARK_INTERFERON_ALPHA_RESPONSE |  | 0.75 | 0.6753 | 0.58 | 0.6753 |
| HALLMARK_SPERMATOGENESIS |  | 0.63 | 0.6753 | 0.67 | 0.6733 |
| HALLMARK_DNA_REPAIR |  | -0.59 | 0.6753 | 0.47 | 0.6753 |
| HALLMARK_NOTCH_SIGNALING |  | -1.57 | 0.0578 | -1.49 | 0.0901 |
| HALLMARK_UV_RESPONSE_UP |  | -1.02 | 0.4123 | -1.08 | 0.2875 |
| HALLMARK_REACTIVE_OXYGEN_SPECIES_PATHWAY |  | -0.95 | 0.4887 | -0.91 | 0.5451 |
| HALLMARK_GLYCOLYSIS |  | 0.71 | 0.6753 | -1.08 | 0.2875 |
| HALLMARK_ADIPOGENESIS |  | -0.71 | 0.6753 | -0.81 | 0.6620 |
| HALLMARK_ESTROGEN_RESPONSE_EARLY |  | -1.13 | 0.1284 | -1.45 | 0.0208 |
| HALLMARK_ANGIOGENESIS |  | -1.03 | 0.4095 | -1.27 | 0.2136 |
| HALLMARK_BILE_ACID_METABOLISM |  | -1.14 | 0.2767 | -1.33 | 0.0938 |
| HALLMARK_MTORC1_SIGNALING |  | -1.34 | 0.0407 | -1.36 | 0.0208 |
| HALLMARK_PEROXISOME |  | -1.25 | 0.1099 | -1.52 | 0.0279 |
| HALLMARK_FATTY_ACID_METABOLISM |  | -1.34 | 0.0597 | -1.55 | 0.0208 |
| HALLMARK_CHOLESTEROL_HOMEOSTASIS |  | -1.08 | 0.3370 | -1.17 | 0.2292 |
| HALLMARK_ANDROGEN_RESPONSE |  | -1.53 | 0.0165 | -1.87 | 0.0012 |
| HALLMARK_MITOTIC_SPINDLE |  | -0.66 | 0.6753 | -1.07 | 0.2875 |
| HALLMARK_ESTROGEN_RESPONSE_LATE |  | -1.33 | 0.0438 | -1.52 | 0.0088 |
| HALLMARK_OXIDATIVE_PHOSPHORYLATION |  | -1.37 | 0.0380 | -1.45 | 0.0202 |
| HALLMARK_MYC_TARGETS_V2 |  | -2.96 | 9E-10 | -2.86 | 9E-10 |
| HALLMARK_MYC_TARGETS_V1 |  | -2.45 | 9E-10 | -2.33 | 9E-10 |
| HALLMARK_G2M_CHECKPOINT |  | -2.37 | 9E-10 | -2.47 | 9E-10 |
| HALLMARK_E2F_TARGETS |  | -2.39 | 9E-10 | -2.55 | 9E-10 |

**Supplementary table 8: Cellular pathways de-enriched and enriched in response to NXP800 treatment in LNCaP95 PCa cells.**

NES – normalized enrichment score, FDR – false discovery rate

|  |  | **22Rv1** | | | |
| --- | --- | --- | --- | --- | --- |
|  |  | **100 nM** | | **250 nM** | |
| **HALLMARK** |  | **NES** | **FDR** | **NES** | **FDR** |
| HALLMARK_UNFOLDED_PROTEIN_RESPONSE |  | 1.31 | 0.1276 | 1.48 | 0.0373 |
| HALLMARK_TNFA_SIGNALING_VIA_NFKB |  | 0.82 | 0.4991 | 1.31 | 0.0692 |
| HALLMARK_HEDGEHOG_SIGNALING |  | 1.43 | 0.0987 | 1.28 | 0.1196 |
| HALLMARK_KRAS_SIGNALING_UP |  | -0.77 | 0.5162 | 0.86 | 0.3705 |
| HALLMARK_TGF_BETA_SIGNALING |  | 0.69 | 0.5162 | 0.93 | 0.3120 |
| HALLMARK_P53_PATHWAY |  | 0.71 | 0.5224 | 0.91 | 0.3541 |
| HALLMARK_HEME_METABOLISM |  | 0.80 | 0.5026 | 0.67 | 0.4668 |
| HALLMARK_HYPOXIA |  | 0.92 | 0.4581 | -1.05 | 0.2099 |
| HALLMARK_COAGULATION |  | -1.05 | 0.3139 | -1.20 | 0.1196 |
| HALLMARK_ALLOGRAFT_REJECTION |  | 1.07 | 0.3139 | -1.06 | 0.2099 |
| HALLMARK_PROTEIN_SECRETION |  | -1.57 | 0.0724 | -0.71 | 0.4618 |
| HALLMARK_INFLAMMATORY_RESPONSE |  | -1.39 | 0.0987 | -1.94 | 0.0004 |
| HALLMARK_IL6_JAK_STAT3_SIGNALING |  | -1.37 | 0.1053 | -1.42 | 0.0511 |
| HALLMARK_MYOGENESIS |  | 0.95 | 0.4101 | -1.02 | 0.2340 |
| HALLMARK_EPITHELIAL_MESENCHYMAL_TRANSITION |  | -1.51 | 0.0370 | -1.79 | 0.0008 |
| HALLMARK_XENOBIOTIC_METABOLISM |  | -1.47 | 0.0494 | -1.31 | 0.0638 |
| HALLMARK_PI3K_AKT_MTOR_SIGNALING |  | 0.60 | 0.5410 | 0.65 | 0.4668 |
| HALLMARK_UV_RESPONSE_DN |  | -1.64 | 0.0186 | -1.23 | 0.0936 |
| HALLMARK_WNT_BETA_CATENIN_SIGNALING |  | 0.67 | 0.5162 | 0.51 | 0.4674 |
| HALLMARK_APICAL_JUNCTION |  | -1.33 | 0.0987 | -1.69 | 0.0019 |
| HALLMARK_APOPTOSIS |  | -1.36 | 0.0945 | -1.05 | 0.2128 |
| HALLMARK_COMPLEMENT |  | -1.60 | 0.0307 | -1.72 | 0.0025 |
| HALLMARK_APICAL_SURFACE |  | -0.83 | 0.4914 | -1.06 | 0.2099 |
| HALLMARK_IL2_STAT5_SIGNALING |  | -1.47 | 0.026 | -1.49 | 0.0116 |
| HALLMARK_PANCREAS_BETA_CELLS |  | -1.38 | 0.0987 | -1.44 | 0.0668 |
| HALLMARK_INTERFERON_GAMMA_RESPONSE |  | -1.44 | 0.0628 | -1.48 | 0.0099 |
| HALLMARK_KRAS_SIGNALING_DN |  | -1.36 | 0.1123 | -2.04 | 7E-05 |
| HALLMARK_INTERFERON_ALPHA_RESPONSE |  | -1.46 | 0.0987 | -1.50 | 0.0295 |
| HALLMARK_SPERMATOGENESIS |  | -2.61 | 2E-05 | -1.44 | 0.0294 |
| HALLMARK_DNA_REPAIR |  | -2.05 | 0.0006 | -1.58 | 0.0065 |
| HALLMARK_NOTCH_SIGNALING |  | 0.68 | 0.5162 | -0.72 | 0.4334 |
| HALLMARK_UV_RESPONSE_UP |  | -0.96 | 0.4101 | -1.23 | 0.0931 |
| HALLMARK_REACTIVE_OXYGEN_SPECIES_PATHWAY |  | 0.39 | 0.5721 | -1.42 | 0.0529 |
| HALLMARK_GLYCOLYSIS |  | -1.12 | 0.1931 | -1.59 | 0.0034 |
| HALLMARK_ADIPOGENESIS |  | -2.06 | 0.0001 | -1.79 | 0.0004 |
| HALLMARK_ESTROGEN_RESPONSE_EARLY |  | -1.47 | 0.0371 | -1.66 | 0.0019 |
| HALLMARK_ANGIOGENESIS |  | -1.46 | 0.0987 | -1.83 | 0.0041 |
| HALLMARK_BILE_ACID_METABOLISM |  | -0.84 | 0.4991 | -1.36 | 0.0529 |
| HALLMARK_MTORC1_SIGNALING |  | -1.77 | 0.0011 | -1.60 | 0.0018 |
| HALLMARK_PEROXISOME |  | -2.00 | 0.0011 | -1.65 | 0.0041 |
| HALLMARK_FATTY_ACID_METABOLISM |  | -2.54 | 2E-06 | -1.87 | 0.0003 |
| HALLMARK_CHOLESTEROL_HOMEOSTASIS |  | -2.45 | 3E-05 | -2.12 | 9E-05 |
| HALLMARK_ANDROGEN_RESPONSE |  | -2.29 | 7E-05 | -1.88 | 0.0004 |
| HALLMARK_MITOTIC_SPINDLE |  | -2.55 | 2E-06 | -1.92 | 4E-05 |
| HALLMARK_ESTROGEN_RESPONSE_LATE |  | -2.29 | 2E-05 | -2.29 | 3E-08 |
| HALLMARK_OXIDATIVE_PHOSPHORYLATION |  | -3.58 | 8E-10 | -2.55 | 8E-10 |
| HALLMARK_MYC_TARGETS_V2 |  | -2.26 | 0.0004 | -2.13 | 9E-05 |
| HALLMARK_MYC_TARGETS_V1 |  | -3.12 | 8E-10 | -2.37 | 2E-09 |
| HALLMARK_G2M_CHECKPOINT |  | -3.57 | 8E-10 | -3.11 | 8E-10 |
| HALLMARK_E2F_TARGETS |  | -4.07 | 8E-10 | -3.37 | 8E-10 |

**Supplementary table 9: Cellular pathways de-enriched and enriched in response to NXP800 treatment in 22Rv1 PCa cells**

NES – normalized enrichment score, FDR – false discovery rate.

|  |  | **NXP800 treated inactive-C 22Rv1 PCa sub-line** | |  |
| --- | --- | --- | --- | --- |
|  |  |  |  |  |
| **HALLMARK** |  | **NES** | **FDR** |  |
| HALLMARK_TNFA_SIGNALING_VIA_NFKB |  | 1.8897408 | 0.0006904 |  |
| HALLMARK_HYPOXIA |  | 1.6039894 | 0.0210139 |  |
| HALLMARK_KRAS_SIGNALING_UP |  | 1.2123262 | 0.2799522 |  |
| HALLMARK_ESTROGEN_RESPONSE_EARLY |  | 1.0792324 | 0.371517 |  |
| HALLMARK_HEDGEHOG_SIGNALING |  | 1.6462888 | 0.0563124 |  |
| HALLMARK_MYOGENESIS |  | 1.4293146 | 0.0675404 |  |
| HALLMARK_P53_PATHWAY |  | 1.5237973 | 0.0354563 |  |
| HALLMARK_UV_RESPONSE_UP |  | 0.8537795 | 0.6391809 |  |
| HALLMARK_HEME_METABOLISM |  | 1.1681701 | 0.2951975 |  |
| HALLMARK_TGF_BETA_SIGNALING |  | 1.3320455 | 0.2097087 |  |
| HALLMARK_XENOBIOTIC_METABOLISM |  | 0.8330599 | 0.6488774 |  |
| HALLMARK_UNFOLDED_PROTEIN_RESPONSE |  | 1.6380078 | 0.0156344 |  |
| HALLMARK_WNT_BETA_CATENIN_SIGNALING |  | 0.6764534 | 0.6981322 |  |
| HALLMARK_PI3K_AKT_MTOR_SIGNALING |  | 0.5635567 | 0.7103522 |  |
| HALLMARK_PANCREAS_BETA_CELLS |  | -0.644753 | 0.6939223 |  |
| HALLMARK_COAGULATION |  | 1.1469629 | 0.3197868 |  |
| HALLMARK_INTERFERON_ALPHA_RESPONSE |  | 1.1643843 | 0.3075965 |  |
| HALLMARK_EPITHELIAL_MESENCHYMAL_TRANSITION |  | -1.149768 | 0.2951975 |  |
| HALLMARK_INTERFERON_GAMMA_RESPONSE |  | 0.9790589 | 0.4899824 |  |
| HALLMARK_APOPTOSIS |  | 1.2421599 | 0.2049353 |  |
| HALLMARK_IL6_JAK_STAT3_SIGNALING |  | -1.180783 | 0.3197868 |  |
| HALLMARK_ESTROGEN_RESPONSE_LATE |  | -1.388797 | 0.0589286 |  |
| HALLMARK_PROTEIN_SECRETION |  | 0.8415707 | 0.6391809 |  |
| HALLMARK_IL2_STAT5_SIGNALING |  | -0.900408 | 0.5997284 |  |
| HALLMARK_MTORC1_SIGNALING |  | -1.140782 | 0.2870813 |  |
| HALLMARK_BILE_ACID_METABOLISM |  | -0.813171 | 0.6391809 |  |
| HALLMARK_ANDROGEN_RESPONSE |  | -1.193097 | 0.2883295 |  |
| HALLMARK_UV_RESPONSE_DN |  | -1.061606 | 0.371517 |  |
| HALLMARK_FATTY_ACID_METABOLISM |  | -1.43286 | 0.0514676 |  |
| HALLMARK_CHOLESTEROL_HOMEOSTASIS |  | -1.684199 | 0.0172559 |  |
| HALLMARK_REACTIVE_OXYGEN_SPECIES_PATHWAY |  | -0.643224 | 0.7099267 |  |
| HALLMARK_ANGIOGENESIS |  | -1.1581 | 0.3333695 |  |
| HALLMARK_ADIPOGENESIS |  | -1.071474 | 0.3663571 |  |
| HALLMARK_INFLAMMATORY_RESPONSE |  | 1.0713153 | 0.371517 |  |
| HALLMARK_KRAS_SIGNALING_DN |  | -1.129349 | 0.3333695 |  |
| HALLMARK_NOTCH_SIGNALING |  | -0.865715 | 0.5997284 |  |
| HALLMARK_PEROXISOME |  | -1.063095 | 0.371517 |  |
| HALLMARK_APICAL_JUNCTION |  | -1.085298 | 0.3663571 |  |
| HALLMARK_GLYCOLYSIS |  | -0.935819 | 0.5796906 |  |
| HALLMARK_SPERMATOGENESIS |  | -1.439532 | 0.0853722 |  |
| HALLMARK_ALLOGRAFT_REJECTION |  | 0.9420836 | 0.5279229 |  |
| HALLMARK_DNA_REPAIR |  | -1.416006 | 0.0589286 |  |
| HALLMARK_MYC_TARGETS_V2 |  | -1.60208 | 0.0378819 |  |
| HALLMARK_MITOTIC_SPINDLE |  | -1.86999 | 0.0001753 |  |
| HALLMARK_APICAL_SURFACE |  | -1.057953 | 0.373378 |  |
| HALLMARK_OXIDATIVE_PHOSPHORYLATION |  | -1.960895 | 3.823E-05 |  |
| HALLMARK_MYC_TARGETS_V1 |  | -1.871543 | 0.0001333 |  |
| HALLMARK_COMPLEMENT |  | -0.974141 | 0.4899824 |  |
| HALLMARK_G2M_CHECKPOINT |  | -2.891505 | 1.895E-09 |  |
| HALLMARK_E2F_TARGETS |  | -3.0423 | 1.895E-09 |  |

**Supplementary table 10: Cellular pathways de-enriched and enriched in response to NXP800 treatment in inactive-C 22Rv1 PCa sub-lines**

NES – normalized enrichment score, FDR – false discovery rate.

|  |  | **NXP800 treated NXP800-R 22Rv1 PCa sub-line** | |
| --- | --- | --- | --- |
|  |  |  |  |
| **HALLMARK** |  | **NES** | **FDR** |
| HALLMARK_TNFA_SIGNALING_VIA_NFKB |  | 1.5466095 | 0.6348921 |
| HALLMARK_HYPOXIA |  | 1.2275246 | 1 |
| HALLMARK_KRAS_SIGNALING_UP |  | 0.9090479 | 1 |
| HALLMARK_ESTROGEN_RESPONSE_EARLY |  | 1.097283 | 1 |
| HALLMARK_HEDGEHOG_SIGNALING |  | 0.9428598 | 1 |
| HALLMARK_MYOGENESIS |  | 0.583594 | 1 |
| HALLMARK_P53_PATHWAY |  | 1.1416755 | 1 |
| HALLMARK_UV_RESPONSE_UP |  | 1.4326671 | 1 |
| HALLMARK_HEME_METABOLISM |  | 1.0281957 | 1 |
| HALLMARK_TGF_BETA_SIGNALING |  | 0.8529937 | 1 |
| HALLMARK_XENOBIOTIC_METABOLISM |  | 1.1889464 | 1 |
| HALLMARK_UNFOLDED_PROTEIN_RESPONSE |  | 1.5552702 | 0.6348921 |
| HALLMARK_WNT_BETA_CATENIN_SIGNALING |  | 0.829736 | 1 |
| HALLMARK_PI3K_AKT_MTOR_SIGNALING |  | 0.7371472 | 1 |
| HALLMARK_PANCREAS_BETA_CELLS |  | 1.1899615 | 1 |
| HALLMARK_COAGULATION |  | -0.509072 | 1 |
| HALLMARK_INTERFERON_ALPHA_RESPONSE |  | -0.658999 | 1 |
| HALLMARK_EPITHELIAL_MESENCHYMAL_TRANSITION |  | 1.092349 | 1 |
| HALLMARK_INTERFERON_GAMMA_RESPONSE |  | -1.008598 | 1 |
| HALLMARK_APOPTOSIS |  | 0.6751836 | 1 |
| HALLMARK_IL6_JAK_STAT3_SIGNALING |  | 1.1039172 | 1 |
| HALLMARK_ESTROGEN_RESPONSE_LATE |  | 0.6941659 | 1 |
| HALLMARK_PROTEIN_SECRETION |  | -0.687628 | 1 |
| HALLMARK_IL2_STAT5_SIGNALING |  | 0.4524469 | 1 |
| HALLMARK_MTORC1_SIGNALING |  | 1.0925603 | 1 |
| HALLMARK_BILE_ACID_METABOLISM |  | 0.514888 | 1 |
| HALLMARK_ANDROGEN_RESPONSE |  | 0.6023919 | 1 |
| HALLMARK_UV_RESPONSE_DN |  | -0.536955 | 1 |
| HALLMARK_FATTY_ACID_METABOLISM |  | 0.537754 | 1 |
| HALLMARK_CHOLESTEROL_HOMEOSTASIS |  | 0.8957557 | 1 |
| HALLMARK_REACTIVE_OXYGEN_SPECIES_PATHWAY |  | 0.9845383 | 1 |
| HALLMARK_ANGIOGENESIS |  | -0.439855 | 1 |
| HALLMARK_ADIPOGENESIS |  | 0.6688671 | 1 |
| HALLMARK_INFLAMMATORY_RESPONSE |  | -1.062986 | 1 |
| HALLMARK_KRAS_SIGNALING_DN |  | -0.948038 | 1 |
| HALLMARK_NOTCH_SIGNALING |  | 0.6593632 | 1 |
| HALLMARK_PEROXISOME |  | 0.807738 | 1 |
| HALLMARK_APICAL_JUNCTION |  | 0.8008448 | 1 |
| HALLMARK_GLYCOLYSIS |  | 0.84961 | 1 |
| HALLMARK_SPERMATOGENESIS |  | -0.85734 | 1 |
| HALLMARK_ALLOGRAFT_REJECTION |  | -1.15107 | 1 |
| HALLMARK_DNA_REPAIR |  | 0.5091866 | 1 |
| HALLMARK_MYC_TARGETS_V2 |  | 0.9805363 | 1 |
| HALLMARK_MITOTIC_SPINDLE |  | -0.29762 | 1 |
| HALLMARK_APICAL_SURFACE |  | -0.446828 | 1 |
| HALLMARK_OXIDATIVE_PHOSPHORYLATION |  | 0.597655 | 1 |
| HALLMARK_MYC_TARGETS_V1 |  | 1.1560718 | 1 |
| HALLMARK_COMPLEMENT |  | -0.718629 | 1 |
| HALLMARK_G2M_CHECKPOINT |  | 0.9501552 | 1 |
| HALLMARK_E2F_TARGETS |  | 0.8438377 | 1 |

**Supplementary table 11: Cellular pathways de-enriched and enriched in response to NXP800 treatment in NXP800-R 22Rv1 PCa sub-lines**

NES – normalized enrichment score, FDR – false discovery rate.

|  |  | **Inactive-C vs NXP800-R 22Rv1 PCa sub-line with inactive chemical control (250 nM CCT365248)** | |  |
| --- | --- | --- | --- | --- |
| **HALLMARK** |  | **NES** | **FDR** |  |
| HALLMARK_TNFA_SIGNALING_VIA_NFKB |  | 1.1635362 | 0.6257504 |  |
| HALLMARK_HYPOXIA |  | 0.8821446 | 0.8738828 |  |
| HALLMARK_KRAS_SIGNALING_UP |  | 1.3617015 | 0.2389758 |  |
| HALLMARK_ESTROGEN_RESPONSE_EARLY |  | 1.3065049 | 0.2944424 |  |
| HALLMARK_HEDGEHOG_SIGNALING |  | 0.8537329 | 0.8738828 |  |
| HALLMARK_MYOGENESIS |  | 1.2870319 | 0.3745851 |  |
| HALLMARK_P53_PATHWAY |  | 0.6220685 | 0.8738828 |  |
| HALLMARK_UV_RESPONSE_UP |  | 0.8777549 | 0.8738828 |  |
| HALLMARK_HEME_METABOLISM |  | 0.7346276 | 0.8738828 |  |
| HALLMARK_TGF_BETA_SIGNALING |  | 0.6989394 | 0.8738828 |  |
| HALLMARK_XENOBIOTIC_METABOLISM |  | 0.6570133 | 0.8738828 |  |
| HALLMARK_UNFOLDED_PROTEIN_RESPONSE |  | -0.666187 | 0.8738828 |  |
| HALLMARK_WNT_BETA_CATENIN_SIGNALING |  | 0.6737066 | 0.8738828 |  |
| HALLMARK_PI3K_AKT_MTOR_SIGNALING |  | 0.685922 | 0.8738828 |  |
| HALLMARK_PANCREAS_BETA_CELLS |  | 1.1882736 | 0.6257504 |  |
| HALLMARK_COAGULATION |  | 1.0578078 | 0.7496431 |  |
| HALLMARK_INTERFERON_ALPHA_RESPONSE |  | 1.1382076 | 0.6257504 |  |
| HALLMARK_EPITHELIAL_MESENCHYMAL_TRANSITION |  | 1.2523963 | 0.4229167 |  |
| HALLMARK_INTERFERON_GAMMA_RESPONSE |  | 1.2019249 | 0.5390034 |  |
| HALLMARK_APOPTOSIS |  | -0.789328 | 0.8738828 |  |
| HALLMARK_IL6_JAK_STAT3_SIGNALING |  | 1.0791117 | 0.73279 |  |
| HALLMARK_ESTROGEN_RESPONSE_LATE |  | 1.3751037 | 0.1856058 |  |
| HALLMARK_PROTEIN_SECRETION |  | 0.4943724 | 0.8738828 |  |
| HALLMARK_IL2_STAT5_SIGNALING |  | 1.0160429 | 0.8187135 |  |
| HALLMARK_MTORC1_SIGNALING |  | 0.5179991 | 0.8738828 |  |
| HALLMARK_BILE_ACID_METABOLISM |  | 0.7422216 | 0.8738828 |  |
| HALLMARK_ANDROGEN_RESPONSE |  | 0.4695209 | 0.8738828 |  |
| HALLMARK_UV_RESPONSE_DN |  | 1.4297979 | 0.1856058 |  |
| HALLMARK_FATTY_ACID_METABOLISM |  | 0.7124892 | 0.8738828 |  |
| HALLMARK_CHOLESTEROL_HOMEOSTASIS |  | 0.502501 | 0.8738828 |  |
| HALLMARK_REACTIVE_OXYGEN_SPECIES_PATHWAY |  | -0.675435 | 0.8738828 |  |
| HALLMARK_ANGIOGENESIS |  | 0.9350524 | 0.8738828 |  |
| HALLMARK_ADIPOGENESIS |  | -0.520469 | 0.8738828 |  |
| HALLMARK_INFLAMMATORY_RESPONSE |  | -1.079909 | 0.6257504 |  |
| HALLMARK_KRAS_SIGNALING_DN |  | 0.9598036 | 0.8738828 |  |
| HALLMARK_NOTCH_SIGNALING |  | -0.997761 | 0.8187135 |  |
| HALLMARK_PEROXISOME |  | -1.012396 | 0.8187135 |  |
| HALLMARK_APICAL_JUNCTION |  | -1.060707 | 0.6882591 |  |
| HALLMARK_GLYCOLYSIS |  | -1.286228 | 0.1856058 |  |
| HALLMARK_SPERMATOGENESIS |  | 0.88626 | 0.8738828 |  |
| HALLMARK_ALLOGRAFT_REJECTION |  | -1.40173 | 0.1916862 |  |
| HALLMARK_DNA_REPAIR |  | -1.515316 | 0.0624609 |  |
| HALLMARK_MYC_TARGETS_V2 |  | -1.961091 | 0.0021259 |  |
| HALLMARK_MITOTIC_SPINDLE |  | -0.465211 | 0.8738828 |  |
| HALLMARK_APICAL_SURFACE |  | -1.170189 | 0.6257504 |  |
| HALLMARK_OXIDATIVE_PHOSPHORYLATION |  | -1.391208 | 0.0688644 |  |
| HALLMARK_MYC_TARGETS_V1 |  | -2.044101 | 8.462E-07 |  |
| HALLMARK_COMPLEMENT |  | -1.171491 | 0.5390034 |  |
| HALLMARK_G2M_CHECKPOINT |  | -1.072724 | 0.6257504 |  |
| HALLMARK_E2F_TARGETS |  | -1.853557 | 2.801E-05 |  |

**Supplementary table 12: Cellular pathways de-enriched and enriched when comparing inactive-C and NXP800-R 22Rv1 PCa sub-lines with inactive chemical control (250 nM CCT365248)**

NES – normalized enrichment score, FDR – false discovery rate.

|  |  | **Castration-resistant VCaP PCa cell line-derived mouse xenograft** | |
| --- | --- | --- | --- |
|  |  |  |  |
| **HALLMARK** |  | **NES** | **FDR** |
| HALLMARK_OXIDATIVE_PHOSPHORYLATION |  | 2.2667808 | 3.60E-05 |
| HALLMARK_ALLOGRAFT_REJECTION |  | 2.237455 | 0.000443 |
| HALLMARK_IL6_JAK_STAT3_SIGNALING |  | 2.1678907 | 0.0034281 |
| HALLMARK_INFLAMMATORY_RESPONSE |  | 1.6906375 | 0.0735544 |
| HALLMARK_KRAS_SIGNALING_UP |  | 1.6866978 | 0.0465202 |
| HALLMARK_INTERFERON_ALPHA_RESPONSE |  | 1.68041 | 0.1001341 |
| HALLMARK_TNFA_SIGNALING_VIA_NFKB |  | 1.6065665 | 0.0508921 |
| HALLMARK_P53_PATHWAY |  | 1.5995014 | 0.0596056 |
| HALLMARK_INTERFERON_GAMMA_RESPONSE |  | 1.52214 | 0.1001341 |
| HALLMARK_FATTY_ACID_METABOLISM |  | 1.4924076 | 0.1001341 |
| HALLMARK_XENOBIOTIC_METABOLISM |  | 1.4846456 | 0.1330999 |
| HALLMARK_EPITHELIAL_MESENCHYMAL_TRANSITION |  | 1.3435001 | 0.1763654 |
| HALLMARK_PROTEIN_SECRETION |  | 1.1884986 | 0.2888596 |
| HALLMARK_DNA_REPAIR |  | 1.1782462 | 0.2888596 |
| HALLMARK_APOPTOSIS |  | 1.1727231 | 0.2888596 |
| HALLMARK_IL2_STAT5_SIGNALING |  | 1.1342816 | 0.3223888 |
| HALLMARK_MYC_TARGETS_V1 |  | 1.1006817 | 0.341854 |
| HALLMARK_UNFOLDED_PROTEIN_RESPONSE |  | 1.0820768 | 0.3760436 |
| HALLMARK_COAGULATION |  | 0.9281826 | 0.5611612 |
| HALLMARK_REACTIVE_OXYGEN_SPECIES_PATHWAY |  | 0.8231697 | 0.6729567 |
| HALLMARK_BILE_ACID_METABOLISM |  | 0.7631588 | 0.7114048 |
| HALLMARK_ADIPOGENESIS |  | 0.7265171 | 0.734856 |
| HALLMARK_ANDROGEN_RESPONSE |  | 0.5940871 | 0.734856 |
| HALLMARK_PEROXISOME |  | 0.4202959 | 0.734856 |
| HALLMARK_UV_RESPONSE_UP |  | -0.350336 | 0.734856 |
| HALLMARK_HEME_METABOLISM |  | -0.403107 | 0.734856 |
| HALLMARK_HYPOXIA |  | -0.448213 | 0.734856 |
| HALLMARK_UV_RESPONSE_DN |  | -0.628549 | 0.734856 |
| HALLMARK_COMPLEMENT |  | -0.692032 | 0.734856 |
| HALLMARK_PANCREAS_BETA_CELLS |  | -0.699327 | 0.7095063 |
| HALLMARK_PI3K_AKT_MTOR_SIGNALING |  | -0.779632 | 0.7095063 |
| HALLMARK_TGF_BETA_SIGNALING |  | -0.887254 | 0.5977585 |
| HALLMARK_MYC_TARGETS_V2 |  | -1.017815 | 0.4744255 |
| HALLMARK_GLYCOLYSIS |  | -1.023046 | 0.4683269 |
| HALLMARK_SPERMATOGENESIS |  | -1.176153 | 0.3223888 |
| HALLMARK_ESTROGEN_RESPONSE_EARLY |  | -1.179976 | 0.3109403 |
| HALLMARK_NOTCH_SIGNALING |  | -1.337486 | 0.2344898 |
| HALLMARK_ESTROGEN_RESPONSE_LATE |  | -1.359227 | 0.1763654 |
| HALLMARK_MTORC1_SIGNALING |  | -1.377189 | 0.1607735 |
| HALLMARK_APICAL_SURFACE |  | -1.401884 | 0.1763654 |
| HALLMARK_KRAS_SIGNALING_DN |  | -1.409367 | 0.1763654 |
| HALLMARK_CHOLESTEROL_HOMEOSTASIS |  | -1.409913 | 0.1763654 |
| HALLMARK_ANGIOGENESIS |  | -1.45161 | 0.1763654 |
| HALLMARK_MYOGENESIS |  | -1.464404 | 0.1581345 |
| HALLMARK_WNT_BETA_CATENIN_SIGNALING |  | -1.691684 | 0.0821448 |
| HALLMARK_HEDGEHOG_SIGNALING |  | -1.769487 | 0.0465202 |
| HALLMARK_APICAL_JUNCTION |  | -1.77266 | 0.0217548 |
| HALLMARK_MITOTIC_SPINDLE |  | -2.588388 | 1.42E-08 |
| HALLMARK_E2F_TARGETS |  | -3.404986 | 1.95E-09 |
| HALLMARK_G2M_CHECKPOINT |  | -3.418168 | 1.95E-09 |
|  |  |  |  |

**Supplementary table 13: Cellular pathways de-enriched and enriched in response to NXP800 treatment in castration-resistant VCaP PCa cell line-derived mouse xenograft**

NES – normalized enrichment score, FDR – false discovery rate.
